# Regional analysis of planting date and cultivar maturity recommendations that improve soybean oil yield and meal protein concentration

**DOI:** 10.3389/fpls.2022.954111

**Published:** 2022-10-17

**Authors:** Montserrat Salmerón, Fred M. Bourland, Normie W. Buehring, Larry Earnest, Felix B. Fritschi, Edward E. Gbur, Bobby R. Golden, Daniel Hathcoat, Josh Lofton, Angela Thompson McClure, Travis D. Miller, Clark Neely, Grover Shannon, Theophilus K. Udeigwe, David A. Verbree, Earl D. Vories, William J. Wiebold, Larry C. Purcell

**Affiliations:** ^1^Department of Plant and Soil Sciences, University of Kentucky, Lexington, KY, United States; ^2^Northeast Research and Extension Center, University of Arkansas, Keiser, AR, United States; ^3^North Mississippi Research and Extension Center, Mississippi State University, Verona, MS, United States; ^4^Southeast Branch Experiment Station, University of Arkansas, Watson, AR, United States; ^5^Division of Plant Science & Technology, University of Missouri, Columbia, MO, United States; ^6^Agricultural Statistics Laboratory, University of Arkansas, Fayetteville, AR, United States; ^7^Delta Research and Extension Center, Mississippi State University, Stoneville, MS, United States; ^8^Texas A&M AgriLife Extension Service, College Station, TX, United States; ^9^Department of Plant and Soil Sciences, Oklahoma State University, Stillwater, OK, United States; ^10^Department of Plant Science, West TN AgResearch Center, University of Tennessee, Jackson, TN, United States; ^11^Department of Plant Sciences, Fisher Delta Research Center, University of Missouri, Portageville, MO, United States; ^12^Department of Plant and Soil Science, Texas Tech University, Lubbock, TX, United States; ^13^Fisher Delta Research Center, United States Department of Agriculture-Agricultural Research Service, Portageville, MO, United States; ^14^Department of Crop, Soil and Environmental Science, University of Arkansas, Fayetteville, AR, United States

**Keywords:** soybean meal, planting date, cultivar maturity group, seed quality, U.S. Midsouth

## Abstract

Planting date and cultivar maturity group (MG) are major management factors affecting soybean [*Glycine max* (L.) Merr.] yield, but their effect on seed oil and protein concentration, and in particular meal protein concentration, is less understood. We quantified changes in seed oil and protein, and estimated meal protein concentration, and total oil and protein yield in response to planting date and cultivar MG ranging from 3 to 6 and across locations comprising a 8.3° range in latitude in the U.S. Midsouth. Our results show that delayed planting date and later cultivar maturity reduced oil concentration, and this was partially associated with a decrease in temperature during the seed fill phase. Thus, optimum cultivar MG recommendations to maximize total oil yield (in kg ha^–1^) for planting dates in May and June required relatively earlier cultivar MGs than those recommended to maximize seed yield. For planting dates in April, short-season MG 3 cultivars did not increase oil yield compared to full-season MG 4 or 5 cultivars due to a quadratic yield response to planting date at most locations. Planting date and cultivar maturity effects on seed protein concentration were not always consistent with the effects on estimated meal protein concentration after oil extraction. Meal protein concentration decreased with lower temperatures during seed fill, and when the start of seed fill occurred after August 15, but relatively short-season cultivar MGs reduced the risk of low meal protein concentration. Meal protein concentration is a trait of interest for the feed industry that would be beneficial to report in future studies evaluating genetic, management, and environmental effects on seed protein concentration.

## Introduction

**FIGURE 1:**
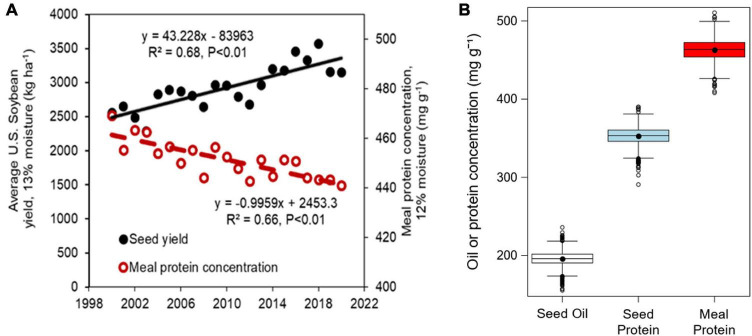
**(A)** Historical U.S. average yield and estimated meal protein concentration during the 2000–2020 period estimated based on Equation 1 with mean U.S. oil and protein concentrations from annual soybean quality reports (Naeve and Miller-Garvin, 2020). **(B)** Boxplots of seed oil and protein concentrations (expressed on a 13 mg g^–1^ moisture basis) and estimated meal protein concentration (expressed on a 12 mg g^–1^ moisture basis) obtained in our study across all years, locations, planting dates, and cultivars.

Soybean meal is the world’s most commonly used source of protein for non-ruminant livestock and poultry. During the period from 2000 to 2020, U.S. soybean yields increased at a rate of 47 kg ha^–1^ yr^–1^ (Naeve and Miller-Garvin, 2019, 2020). However, seed and meal protein concentrations declined by 0.79 and 1 mg g^–1^ yr^–1^ during the same period, respectively (Figure 1A). Maintaining the meal protein concentration and overall quality of soybean meal is essential for proper development of poultry and livestock fed from soybean meal, and for ensuring the competitivity of U.S. soybean in national and international markets. The U.S. National Oil Processors Association requires a minimum of 440 mg protein g^–1^ (120 mg g^–1^ moisture basis) in non-dehulled soybean meal, or 475 mg protein g^–1^ (120 mg g^–1^ moisture basis) in dehulled soybean meal after oil extraction for high protein meal designation (NOPA, 2021). Soybean produced in the Northern U.S. states usually have lower protein concentration than those grown in the south (Wilcox et al., 1979; Voldeng et al., 1997; Chung et al., 2003; Rotundo et al., 2016). However, there is high inter-annual and within state variability in seed oil and protein concentration that is as large or more than the variation reported across different US states (Rotundo et al., 2016). Part of this variation can be attributed to genetic, environmental, and management factors (Yaklich et al., 2002; Rowntree et al., 2013), but achieving a better understanding of the interactive role of these factors is still needed. In addition, research efforts evaluating the interactive effects of genotype, environment, and management factors on seed composition have focused on seed oil and protein concentration (Mourtzinis et al., 2017; Assefa et al., 2019) and less on meal protein concentration (Mourtzinis et al., 2018).

Part of the decreasing trend in U.S. seed protein concentration can be explained by modern soybean cultivars released in the US having lower seed protein concentration and higher yield on average compared to cultivars released earlier and among ancestors (Mahmoud et al., 2006). Some genetic differences in seed protein concentration within modern cultivars may be associated with differences in cultivar maturity (Yaklich et al., 2002). There is little information whether cultivars of different maturity but of similar yield potential could provide an opportunity to increase seed and meal protein concentrations. For instance, cultivar maturities from early MG 4 to late 5 provided the highest and similar yields for early planting dates in the U.S. Midsouth, whereas cultivar MG from early 3 to late 4 maximized yield for relatively late planting dates (Salmerón et al., 2014, 2016).

In addition to genetic factors, environmental conditions also play an important role in determination of soybean seed composition. The quadratic relationship between seed oil concentration and temperature during seed growth is well documented (Piper and Boote, 1999; Thomas et al., 2003; Carrera et al., 2009), as well as the interactive effect of water stress on this relationship (Dornbos and Mullen, 1992; Carrera et al., 2009). In contrast, the relationship between temperature during seed growth and seed protein concentration is more inconsistent. Protein concentration decreased with maximum temperatures during seed fill in the study conducted by Robinson et al. (2009) in Indiana. In other studies, protein concentration decreased or had no significant response to temperature during seed fill (Weiss et al., 1952; Howell and Cartter, 1958; Serretti, 1993; Kane et al., 1997; Naeve and Huerd, 2008). Some studies found an inverse quadratic relationship between seed protein concentration and average temperature during seed fill (Piper and Boote, 1999; Thomas et al., 2003; Carrera et al., 2009; Alsajri et al., 2020), that might be partially due to the indirect effect of temperature on seed oil concentration. Previous studies did not analyze the effect of temperature on meal protein concentration after oil extraction.

Management factors such as planting date could modify seed composition through an indirect effect on environmental conditions and on the crop yield potential. The negative relationship between yield and seed protein concentration of modern high yielding cultivars grown across different environments and management practices is well known (Ortez et al., 2018; La Menza et al., 2019). Early planting dates can increase soybean yield, whereas yield is delayed after an optimum planting window (Egli and Cornelius, 2009; Salmerón et al., 2017), which may cause a diluting or concentrating effect, respectively, on the seed protein concentration. Studies evaluating the effect of planting date on seed composition found that protein concentration often increases, and oil concentration declines when planting date is delayed (Pendleton and Hartwig, 1973; Kane et al., 1997; Heatherly and Elmore, 2004; Bastidas et al., 2008; Robinson et al., 2009; Rowntree et al., 2013). However, in another planting date study, delayed planting reduced protein concentration in the U.S. Midsouth (Jaureguy et al., 2013). Research evaluating the effect on seed protein concentration of early planting dates that maximize yield potential is still limited. In addition, there is a wide range of soybean maturity group (MG) choices usually well-adapted within a region that may respond differently to planting date, providing an opportunity to increase yield without detrimental effects on seed protein concentration.

We analyzed seed oil and protein and estimated meal protein concentration from a regional study conducted in nine U.S. Midsouth locations comprising a 8.3° range in latitude over 3 years (2012–2014), with four planting dates at each location, and cultivar maturities ranging from MG 3 to 6 at each site (Salmerón et al., 2016). Our specific objectives were (i) to analyze the variability in seed oil and protein concentration, and meal protein concentration in response to planting date, cultivar MG, and variation in temperature during seedfill, and (ii) to identify optimum management recommendations that maximize total oil and protein yield, and compare them with optimum management recommendations reported by Salmerón et al. (2016) for the same experimental data. An expected outcome from this study is to provide planting date and cultivar maturity recommendations that may maximize oil yield and reduce risk of low meal protein concentration.

## Materials and methods

### Description of field experiments

A multi-environment planting date and MG trial was conducted at seven locations in the U.S. Midsouth during 2012 (30.6–36.4°N) and nine locations in 2013 and 2014 (30.6–38.9°N). Details from the locations, experimental design, and methods can be found in Salmerón et al. (2016). Briefly, the experimental design within each location was a split-plot with four replicates, planting date as the main factor, and four different planting dates ranging from late March to early July (Table 1). Cultivar MG was the split-plot factor, with MG 3–6, and with four cultivars nested randomly within each MG. The same 16 commercial cultivars were used each year across locations, however, some cultivars were modified from year to year and replaced by cultivars of similar maturity.

**TABLE 1:** Planting dates at each location and year grouped in Early (before May 1), May planting dates, and Late (after May 30).

Location (Latitude)	Early planting (before May 1)	May planting	Late planting (after May 30)
**2012**			
Portageville, MO (36.4 N°)	April 2, April 17	May 10	June 12
Keiser, AR (35.7 N°)	March 30, April 19	May 16	June 8
Verona, MS (34.2 N°)	March 21, April 11	May 17	June 6
Rohwer, AR (33.8 N°)	March 29, April 24	May 15	June 26
Stoneville, MS (33.4 N°)	March 20, April 13	May 10	June 7
St Joseph, LA (32.0 N°)	April 6, April 20	May 15	June 1
College Station, TX (30.6 N°)	March 26, April 12	May 4	May 25^†^
**2013**			
Columbia, MO (38.9 N°)	May 8^‡^	May 14	June 4, June 25
Portageville, MO (36.4 N°)	April 9	May 9, May 29	June 20
Milan, TN (35.9 N°)	April 22	May 9	June 5, June 25
Keiser, AR (35.7 N°)	–	–	June 13, 26, July 8, 17
Verona, MS (34.2 N°)	April 23	May 15, May 30	June 17
Rohwer, AR (33.8 N°)	April 26	May 20	June 10, June 28
Stoneville, MS (33.4 N°)	April 18	May 31	June 12, June 27
St. Joseph, LA (32.0 N°)	April 29	May 14, May 28	June 12
College Station, TX (30.6 N°)	April 9, April 26	May 13	May 30^†^
**2014**			
Columbia, MO (38.9 N°)	April 23	May 21	June 17, June 27
Portageville, MO (36.4 N°)	April 22	May 7, May 27	June 17
Milan, TN (35.9 N°)	April 24	May 7	June 17, July 3
Keiser, AR (35.7 N°)	April 23	May 8, May 22	June 5
Verona, MS (34.2 N°)	April 23	May 13, May 27	June 17
Rohwer, AR (33.8 N°)	April 21	May 19	June 5, June 30
Stoneville, MS (33.4 N°)	–	May 8, May 23	June 6, July 2
St. Joseph, LA (32.0 N°)	April 24	May 8, May 22	June 19
College Station, TX (30.6 N°)	April 9, April 25	May 12	June 2

^†^Last planting dates occurred in late May and were considered already a late planting date at this warm location.

^‡^The first planting date occurred in May 8 but was considered an Early planting date at this relatively cool location.

Plots were 6 m long and had four single or twin rows, depending on the year and location. Row spacing ranged from 38 to 76 cm in single rows. Twin rows were planted on beds spaced 97 cm apart to facilitate furrow irrigation; the spacing between rows on a bed ranged from 19 to 48 cm depending on the location. Seeding rate was 35 m^–2^. All experiments were irrigated when the cumulative net evapotranspiration demand reached values of 30–50 mm, depending on soil characteristics at each location. Daily minimum and maximum air temperature data were obtained from weather stations located onsite or downloaded from the National Oceanic and Atmospheric Administration, Climate Data Online tool, within a 3-mile radius from the experimental trials. Dates of developmental stages were recorded as described in Salmerón and Purcell (2016). The average daily air temperature during the seed filling phase (T_*R*5–*R*7_) was calculated from the R5 to R7 stages as defined by Fehr and Caviness (1977). Yield (kg ha^–1^) was measured by harvesting 4.9 to 6 m of the two central rows of each plot (4.6–9.6 m^2^ in total depending on the location), and is reported at 130 mg g^–1^ water basis.

A seed subsample from each plot was sent to the Missouri Agricultural Experiment Station Fisher Delta Research Center Seed Quality Laboratory (Portageville, MO, United States) to analyze oil and protein concentration (mg g^–1^) by near-infrared spectroscopy (NIRS) technology (Infratec 1255 Grain Analyzer, Foss Instruments, Eden Prairie, MN, United States), with calibration equations developed by Foss Instruments (Calibration number SO981011). Seed oil and protein concentrations were adjusted to a 130 mg g^–1^ moisture basis. Absolute amounts of oil and protein on an area basis (kg ha^–1^) were calculated as the product of yield and oil or protein concentration and expressed on a dry weight basis. The protein concentration in meal after the extraction of oil was estimated using equation 1 based on Brumm and Hurburg (1990). This equation assumes that soybean seed is processed at 130 mg g^–1^ moisture, that seed has a test weight of 772 g L^–1^ (60 lb bu^–1^), that there is a 1.15% total dry matter loss in the crushing process, a residual oil concentration in the meal of 12 mg g^–1^, and the meal moisture is 120 mg g^–1^.


(1) $$Meal\;protein\;concentration\;(mgg^{-1})\;=-\mathrm{0.1343}+\mathrm{0.6712}Oil+\mathrm{1.3203}Protein$$

Where *Oil* and *Protein* are concentration in mg g^–1^ of oil and protein in whole seeds with 130 mg g^–1^ moisture, respectively. This approach to estimate meal protein concentration was previously used by others (e.g., Mourtzinis et al., 2018; Chiluwal et al., 2021) and provided similar values compared to calculating meal protein concentration by subtracting oil concentration (*y* = 0.9967x−2.7557, *R*^2^ = 0.99) but 0.2–1.5% lower due to inefficiencies in the crushing and oil extraction process.

The yield, oil, and protein concentration data from this study in the US Midsouth was previously analyzed by Assefa et al. (2019) as part of a larger national dataset. In this study we further analyzed seed oil and protein concentration, as well as estimated meal protein concentration and oil and protein yield, considering the well-balanced experimental design evaluating planting date and cultivar MG factors and their interactions that were not considered fully in the study by Assefa et al. (2019).

### Data analysis

#### Analysis of variance

An analysis of variance was done using the PROC MIXED procedure in SAS (SAS v.9.4, SAS Institute, Inc., Cary, NC, United States) to quantify the relative contribution of each considered factor to the total variability of oil and protein concentration, meal protein concentration, and total oil and protein yield. The analysis of variance included year, location, planting date, MG, cultivars (nested within MG and year), and their interactions as fixed effects. Blocks were grouped in two sets, where blocks within a set shared the same planting date and MG randomization to facilitate planting and harvest operations. Random effects considered in the model were set × year × location, block × set × year × location, and their interactions with planting date and cultivar MG. The sum of squares from the ANOVA were grouped by sources of variation related to environment (year, location, planting date and their interactions), genotype (MG and cultivar within MG and year), and environment x genotype (all possible combinations across the previous factors). The percentage of sum of squares in the model explained by each group was calculated dividing the sum of squares explained by each source of variation by the total sum of squares in the model, and multiplying by 100.

#### Analysis of seed oil, protein, and estimated meal protein concentration data

##### Effect of early and late planting dates

Each location included four planting dates that changed from year to year depending on spring precipitation patterns at each location. Thus, planting dates were grouped in Early (planting dates before May 1), May (planting dates during May), and Late (Planting dates after May 30) to provide a better interpretation of the effect of planting to our analysis. This grouping resulted in 1–2 planting dates within each group (Early, May, Late), with few exceptions (Table 1). The effect of Early and Late planting dates (relative to planting dates in May) on seed oil and protein concentration, and on estimated meal protein concentration was analyzed with the above-mentioned ANOVA using PROC MIXED in SAS (see section “Analysis of variance”). The lsmestimate statement was used to do custom hypothesis tests for each year, location, and MG combination, and obtain the estimate of the difference between the mean seed oil and protein concentrations across planting dates in May, with the mean across planting dates before May or after May. We expressed the effect of planting Early or Late as the difference in oil or protein concentrations under Early or Late planting, minus the oil or protein concentrations under planting dates in May, and this effect was considered significantly different from 0 at *P* < 0.05.

##### Effect of cultivar maturity

To analyze the effect of genotype that may be associated to changes in cultivar maturity, we analyzed the rate of change in seed oil, protein, and estimated meal protein concentration in response to the cultivar relative maturity group (rMG). Seed oil, protein, and meal protein concentration data were analyzed with a linear regression model that was dependent on the location, year, and planting date. An analysis of covariance with PROC MIXED in SAS (SAS v.9.4, SAS Institute, Inc., Cary, NC, United States) was used, that included rMG as a covariable, and location, year, planting period (Early, May, and Late), and their interaction as fixed factors allowed to modify the response to rMG. Data were averaged across replicates before this analysis.

##### Relationship with day of R5 and temperature during seedfill

We found that variability in seed oil, seed protein, and estimated meal protein concentration was best explained by a bilinear model with day of beginning seed (R5) as the independent variable compared to using planting day of year or a quadratic model fit. The PROC NLIN procedure in SAS was used to fit a bilinear model of seed oil, protein concentration, and estimated meal protein concentration in response to date of R5. The data were analyzed across locations separated in two groups based on their latitude (locations at latitudes below 35°N and above 35°N), following a similar approach to Egli and Cornelius (2009) and Assefa et al. (2019) when evaluating the soybean yield response to planting date across a wide range of latitudes. We also analyzed the variability in seed oil, protein, and estimated meal protein concentration associated with changes in T_*R*5–*R*7_. The PROC MIXED procedure was used to fit a quadratic model of seed oil, protein concentration, and meal protein concentration in response to T_*R*5–*R*7_, and with soybean cultivar as random effect in the model. Data were analyzed across all locations (and not by latitude group) to provide a wider range of temperatures to fit our models. The partial *R*^2^ values were used to quantify the variability in seed oil, seed protein, and meal protein concentration explained by T_*R*5–*R*7_ or cultivar. Data were averaged across replicates by treatment before fitting the models.

#### Analysis of total oil and protein yield

The effect of planting date and cultivar MG on total oil and protein yield (in kg ha^–1^) and on seed yield was analyzed by fitting the data to a quadratic or linear model in response to planting day of year (PDOY) for each location, year, and cultivar MG. We analyzed total oil and protein yield data with the relationship with PDOY to be consistent with approaches in previous studies analyzing the seed yield response to PDOY (Egli and Cornelius, 2009; Salmerón et al., 2016), and given that PDOY explained a relatively larger part of the variation in total oil and protein yield, compared to the other variables analyzed in this study. Data were averaged across replicates before analyzing the response to PDOY. An analysis of covariance using the PROC MIXED procedure (SAS, v.9.4, SAS Institute, Inc., Cary, NC, United States) was used to test the effect of location and MG on the shape of the oil and protein yield response to PDOY. Location, MG, and their interaction were included as fixed effects in the model. The PDOY and its square were included in the model as independent variables, as well as their interaction with all fixed effects. The year nested within location, and the cultivar nested within MG and year were included as random effects in the model. A linear model was used when the quadratic term of the regression was not significant. The shape of the relationship was specified for each location and MG combination in the model by multiplying the square of PDOY by a constant with a value of 0 or 1. The dispersion of the observed data from the model fit was quantified from the root mean square error (RMSE) of the residuals from the observed data with the model estimates for each location and MG combination. The optimum planting dates that maximized total oil and protein yield by location and MG were obtained from the estimated model fits. We also estimated seed yield, protein yield, and oil yield from the fitted models at three different planting dates (April 15, May 15, and June 15) to test differences across MGs within a location and compare them with those by Salmerón et al. (2016). The lsmeans statement was used to compute least square means within a location and MG at a given PDOY (April 15, May 15, or June 15), and differences across means were tested with the PDIFF option and considered different at *P* < 0.05. To present data in figures and tables, estimates of seed yield, protein yield, and oil yield data were normalized dividing by the highest yield achieved within a location based on the model fits by cultivar MG.

## Results

### Analysis of sources of variation

**FIGURE 2:**
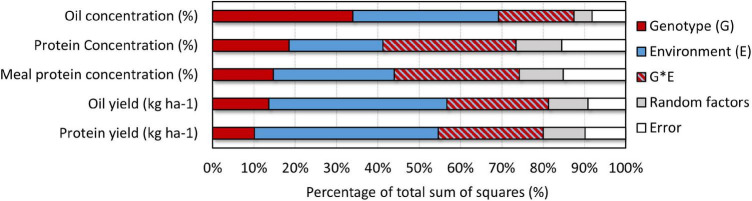
Summary of effects explaining the variability in seed oil and protein concentration, and in estimated meal protein concentration combining sources of variation related to environment (E), genotype (G), and the G × E interaction based on the ANOVA analysis (Table 2). Sources of variation related to environment are location, year, planting date, and their interaction. Sources of variation related to genotype are cultivar maturity group (MG), and cultivars nested within MG and year. Sources of variation related to the G × E interaction include all the interactions from the abovementioned effects.

The seed oil and protein concentrations, and estimated meal protein concentration averaged 196, 353, and 463 mg g^–1^, respectively, and showed large variability across the treatments, locations, and years in our study (Figure 1B). The analysis of variance on seed oil, seed protein, and estimated meal protein concentration revealed a significant effect from all factors and interactions (Table 2). The grouped sources of variation related to environment explained a relatively large percentage of the variability in seed oil and protein concentrations, and in meal protein concentration (23–35%, Figure 2). However, the variability in seed oil concentration due to environment was mainly associated with a location effect (22%), whereas seed and meal protein concentration were most variable due to year (7–11%) and the year-by-location interaction (5–7%, Table 2). Of interest, genotype explained a large part of the variation in seed oil concentrations (34%), but relatively less of the variation in seed protein concentration (19%) and meal protein concentration (15%) (Figure 2). The percentage of variability explained by the genotype by environment interaction was lowest for seed oil concentration (18%), and increased for seed protein and meal protein concentrations (30–32%). Similarly, unaccounted sources of variability not explained by fixed or random factors included in the ANOVA model were lowest for seed oil concentration (8%), and relatively greater for seed protein (16%), and meal protein concentration (15%, Figure 2).

**TABLE 2:** Summary of fixed factors from the analysis of variance of seed oil and protein concentration, estimated meal protein concentration, and total oil and protein yield.

Source of variation	DF^†^	Pr > F	% sum of squares in the model				
			Seed oil (%)	Seed protein (%)	Meal protein (%)	Oil yield (kg ha^–1^)	Protein yield (kg ha^–1^)
Location (L)	8	<0.001	22.0	1.9	2.2	19.1	22.1
Year (Y)	2	<0.001	0.6	7.0	10.7	0.7	0.4
Y * L	14	<0.001	4.7	5.2	7.2	9.5	9.7
Planting (PD)	3	<0.001	4.0	1.6	0.4	7.7	6.0
L * PD	24	<0.001	1.3	3.2	4.2	2.0	2.1
Y * PD	6	<0.001	0.5	0.3	0.4	0.3	0.4
Y * L * PD	42	<0.001	2.1	3.4	4.1	3.7	3.8
Maturity group (MG)	3	<0.001	22.1	3.0	0.2	11.8	8.0
L*MG	24	<0.001	2.8	6.1	7.2	3.4	3.9
PD*MG	9	<0.001	0.2	0.6	0.4	1.1	0.9
L*PD*MG	72	<0.001	1.3	2.7	2.4	2.4	2.6
Y*MG	6	<0.001	0.7	0.7	0.4	0.7	0.5
Y*L*MG	42	<0.001	1.9	2.8	2.9	2.5	2.4
Y*PD*MG	18	<0.001	0.2	1.1	1.2	1.1	1.0
Y*L*PD*MG	122	<0.001	1.8	3.8	3.8	3.7	4.0
Cultivar(Y*MG)	36	<0.001	11.9	15.5	14.5	1.8	2.1
L*Cultivar(Y*MG)	263	< 0.001	4.6	5.8	4.4	4.1	4.1
PD*Cultivar(Y*MG)	108	<0.001	1.1	2.0	1.7	0.9	0.9
L*PD*Cultivar(Y*MG)	754	<0.001	3.6	6.7	5.8	4.6	4.9

^†^DF, degrees of freedom.

The total oil and protein yield (kg ha^–1^) differed in the amount of variability explained by different factors in the ANOVA compared to the analysis of seed oil, seed protein, and estimated meal protein concentration (Table 2). Environment explained the highest percentage of the variability for total oil and protein yields (43–45%), followed by the genotype by environment interaction (25%), and genotype explained the lowest percentage of the variability in total oil and protein yields (10–14%) (Figure 2). The genotype effect on total oil and protein yield was mostly due to a MG effect (8–12%) rather than cultivar (2%) (Table 2).

### Analysis of seed oil and protein concentration, and estimated meal protein concentration

#### Effect of early and late planting dates

**FIGURE 3:**
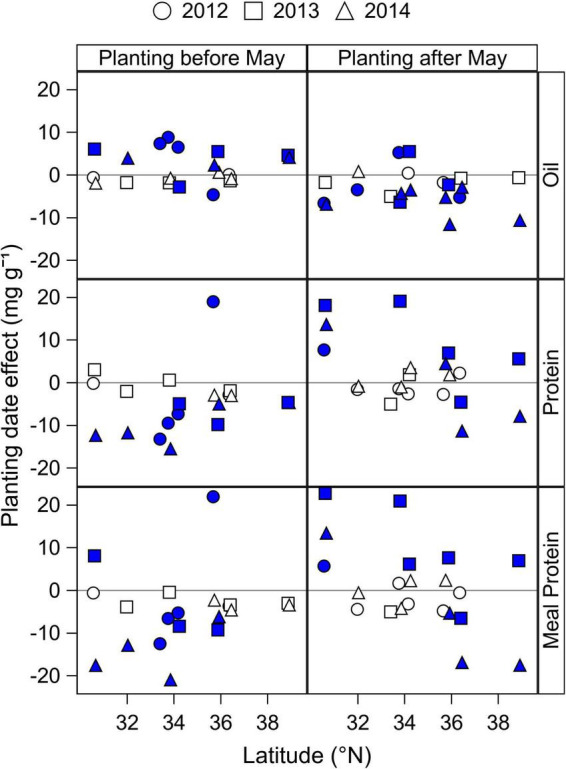
Mean effect of Early (before May) and Late (after May) planting dates by latitude and year on seed oil and protein concentration, and on estimated meal protein concentration of MG 4 cultivars. Closed symbols indicate a significant planting date effect at *P* < 0.05, obtained from the ANOVA analysis (Table 2). The planting date effect was calculated as the difference in seed and meal composition of a cultivar MG grown before or after May minus the seed and meal composition of the same cultivar MG grown in May within each year and site. Positive values indicate a positive effect of planting before or after May, relative to planting dates in May. Negative values indicate a negative effect of planting before or after May, relative to planting dates in May. Results from cultivars of MG 3, 5, and 6 are provided in Supplementary materials (Supplementary Figures 2–4).

The effect of Early and Late planting dates, relative to planting dates in May, on seed oil, protein, and estimated meal protein concentrations is shown in Figure 3 for MG 4 cultivars, and the same analysis is provided in Supplementary materials (Supplementary Figures 1–3) for MG 3, 5, and 6 cultivars. Figure 3 shows results from MG 4 cultivars since this was the optimum MG choice recommendation to maximize yield across planting dates in most locations based on the yield analysis from the same dataset by Salmerón et al. (2016). Planting dates before May increased seed oil concentration compared to planting in May in nine out of 24 site-years (2.3–8.8 mg g^–1^ increase, difference from the 0 horizontal line in Figure 3) and on average by 1.7 mg g^–1^ across locations and years (Figure 3). In contrast, when planting date was delayed after May, oil concentration was reduced in 13 site-years (2.3–11.6 mg g^–1^ decrease), and on average by 3.3 mg g^–1^ across locations and years (Figure 3).

Seed protein concentration had a tendency to decrease on average with planting dates before May by 4.4 mg g^–1^ compared to planting dates in May, with a significant reduction of 4.6–13.2 mg g^–1^ in 10 site-years (Figure 3). One exception was the site at Keiser, AR in 2012 that had a significant increase in seed protein concentration when planting before May. Delaying planting dates after May had a variable effect on seed protein concentration that depended on the location and year, ranging from 4 to 19 mg g^–1^ increase in seven site-years, to 5–11 mg g^–1^ reduction in three site-years compared to planting dates in May.

Early planting dates before May reduced the estimated meal protein concentration on average by 4.7 mg g^–1^), with a significant reduction in nine site-years (5–20 mg g^–1^ decrease) and a significant increase in two site-years (5–20 mg g^–1^ increase) (Figure 3). When planting dates were delayed after May, meal protein concentration increased by 6–22 mg g^–1^ in six site-years, and decreased by 5–17 mg g^–1^ in four site-years. Overall, delaying planting dates after May had a tendency to increase seed and meal protein concentrations in relatively southern latitudes within our study, but it decreased meal protein concentration in some locations and years in northernmost latitudes.

The analysis of planting date effect on seed oil, seed protein, and estimated meal protein concentration of MG 3, 5, and 6 cultivars (Supplementary Figures 1–3) showed parallel results to those observed for MG 4 cultivars, with some exceptions for early planting dates. Advancing planting dates before May had a mixed effect on seed oil concentration of short-season MG 3 cultivars (Supplementary Figure 1), but was more likely to increase oil concentration in full-season MG 4 and 5 cultivars (Figure 3 and Supplementary Figure 2). For seed and meal protein concentrations, advancing planting date before May with MG 5 cultivars showed a clear negative effect that was most pronounced with decreasing latitude (Supplementary Figure 2).

#### Effect of cultivar maturity

**FIGURE 4:**
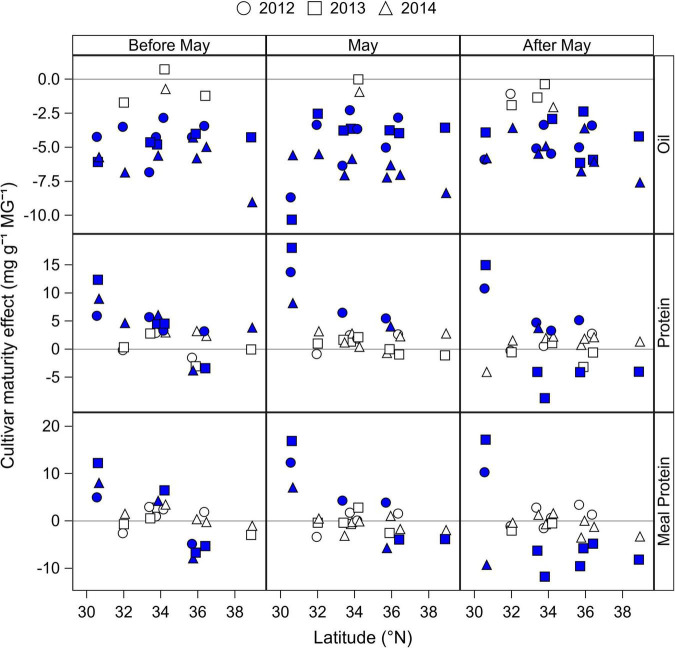
Change in seed oil and protein concentrations, and in estimated meal protein concentration with increase in cultivar maturity (mg g^–1^ rMG^–1^) by latitude and year, and for planting dates before May, in May, and after May. Data obtained from the slope of the regression of seed oil and protein concentrations, and estimated meal protein concentration with cultivar relative maturity group (rMG) by year, location, and planting period (before May, May, after May). Filled symbols indicate a slope significantly different from zero at *P* < 0.05 based on the ANCOVA analysis (Table 3). Positive and negative values indicate an increase and decrease, respectively, on the average seed oil and protein concentration, or meal protein concentration within a location and year when switching to cultivars of longer maturity.

The effect of cultivar maturity by planting date was analyzed by fitting seed oil and protein concentrations, and estimated meal concentration to a linear regression model as a function of rMG that was dependent on the location, planting date, and year (Table 3). From the analysis of covariance model, the effect of cultivar maturity, measured as the rMG main effect (slope of the regression) or its interaction with location, year, and planting date explained 46% of the variability in oil concentration, but relatively less of the variability in seed and meal protein concentrations (21–24%) (Table 3). Given that the seed protein and meal protein concentrations response to rMG was dependent on the location, year, and planting date, data obtained from the slope of the regressions are presented in Figure 4 by latitude, year, and planting date. Seed oil concentration decreased in 40 out of the 48 site-planting time-year combinations when switching to later maturities at a rate 2.9–9.0 mg g^–1^ rMG^–1^, and by 4.3 mg g^–1^ rMG^–1^ on average (Figure 4). In contrast, the cultivar maturity effect on seed protein and meal protein concentrations was variable depending on the planting date, location, and year (Figure 4). However, some clear trends associated with planting date and latitude were observed. For instance, delaying cultivar maturity in planting dates before May had the most chances of increasing seed protein concentration (significant increase of 3.8–12.6 mg g^–1^ rMG^–1^ in 11 out of 20 site-years), compared to planting dates in May (6 out of 20 cases) or after May (6 out of 20 cases). Of interest, delaying cultivar maturity increased seed protein concentration in 23 out of 60 site-planting date-year combinations, but was less effective increasing meal protein concentration (12 cases across all planting dates). Lastly, delaying cultivar maturity was more likely to increase meal protein concentration at relatively southern locations in our study (significant increase in 11 site-planting time-year combinations for latitudes < 35 °N), compared to northernmost locations (1 site-planting time-year combination for latitudes > 35 °N). Instead, delaying cultivar maturity showed a tendency to decrease meal protein concentration in latitudes < 35 °N (significant decrease in 11 site-planting time-year combinations).

**TABLE 3:** Analysis of covariance for seed oil and protein concentration (mg g^–1^), and estimated meal protein concentration (mg g^–1^) with rMG as independent variable, and location, year, and planting period (Early, May, or Late) allowed to affect the intercept and slope of the regression.

		Oil concentration (%)			Protein concentration (%)			Meal protein concentration (%)		
Source	DF^†^	*F*-value	Pr > F	% sum of squares	*F*-value	Pr > F	% sum of squares	*F*-value	Pr > F	% sum of squares
L*Y*PD	71	2.88	<0.001	9.3	5.55	<0.001	21.7	6.06	<0.001	23.9
rMG	1	812.27	<0.001	36.8	96.58	<0.001	5.3	0.14	0.7116	0.0
rMG*L*Y	24	5.85	<0.001	6.4	10.39	<0.001	13.7	12.16	<0.001	16.2
rMG*PD	2	2.31	0.0993	0.2	5.8	0.0031	0.6	5.45	0.0044	0.6
rMG*L*Y*PD	45	1.19	0.1824	2.4	1.62	0.0064	4.0	1.77	0.0016	4.4
Residual	990	–	–	44.9	–	–	54.6	–	–	54.9

^†^DF, degrees of freedom.

#### Relationship with day of R5 and temperature during seedfill

**FIGURE 5:**
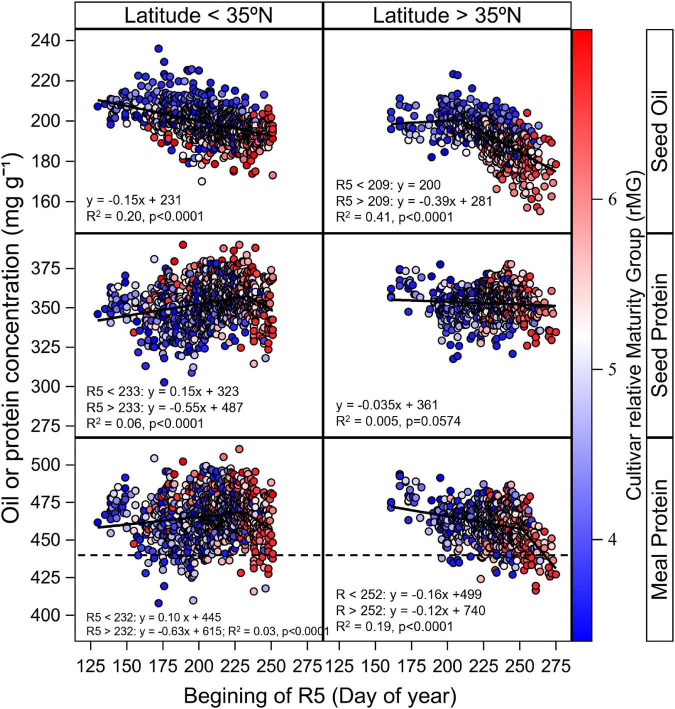
Relationship between seed oil and protein concentration and estimated meal protein concentration with day of beginning seed (R5) across sites with latitude above and below 35°N within our study. Data symbols represent data observed data by cultivar averaged across replicates. The solid line indicates the fitted bilinear model obtained with PROC NLIN for the analysis of seed oil, protein, and estimated meal protein concentration with day of R5 as independent variable. The bilinear or linear model fits from the analysis are shown in the figures. The gradient colors in data symbols represent the cultivar relative Maturity Group (rMG). The dashed horizontal line represents the minimum meal protein concentration for high protein meal designation. Data for seed and oil protein concentration presented on a 13 mg^–1^ g moisture basis, and data from estimated meal protein concentration is presented on a 12 mg g^–1^ moisture basis.

Results shown in Figures 3, 4 indicated a clear interactive effect of latitude, planting date, and cultivar maturity on seed and estimated meal protein concentration. This response may be partially associated with different timing of the start of beginning seed (R5), and with T_*R*5–*R*7_. The relationship between seed oil concentration and date of R5 (Figure 5) showed that oil concentration decreased as the date of R5 was delayed at a rate of 0.15 mg g^–1^ day^–1^ in latitudes below 35°N (*p* < 0.001, *R*^2^ = 0.20), and at a rate of 0.39 mg g^–1^ day^–1^ when date of R5 was delayed after the month of July in latitudes above 35°N (*p* < 0.001, *R*^2^ = 0.41). Only a small part of the variability in seed protein concentration was associated with changes in the date of R5 (Figure 5). Seed protein concentration increased at a rate of 0.15 mg g^–1^ day^–1^ when date of R5 was delayed up to the end of August in latitudes below 35°N (*p* < 0.001, *R*^2^ = 0.06), and did not respond to changes in date of R5 for latitudes above 35°N (Figure 5). In contrast, meal protein concentration decreased in latitudes above 35°N when date of R5 was delayed at a rate of 0.63 mg g^–1^ day^–1^, and at a rate of 1.12 when date of R5 was delayed past early September (*p* < 0.001, *R*^2^ = 0.19) (Figure 5).

**FIGURE 6:**
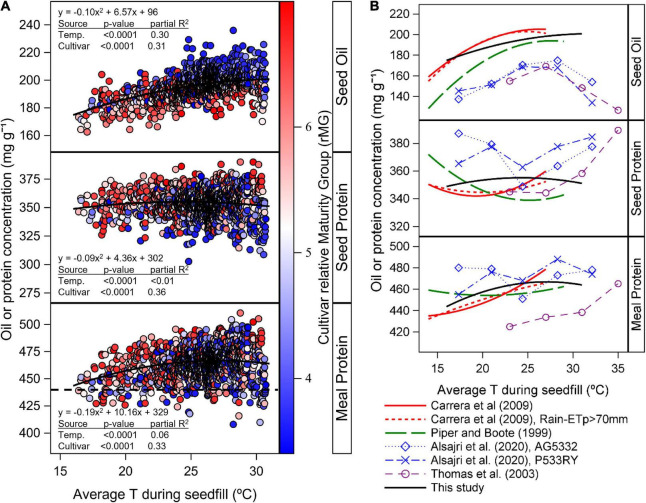
**(A)** Relationship between seed oil and protein concentration and estimated meal protein concentration with temperature during seedfill (*T*_R5–R7_) across all cultivars, planting dates, and sites. Data symbols represent observed data by cultivar and averaged across replicates. Solid lines represent the quadratic model fit for the response of seed oil and protein concentration, and estimated meal protein concentration with *T*_R5–R7_ as independent variable and with cultivar as random factor in the model. The gradient colors in data symbols represent the cultivar relative Maturity Group (rMG). The dashed horizontal line indicates the minimum meal protein concentration for high protein meal designation. **(B)** Literature review of the relationship between seed oil and protein concentration, and estimated meal protein concentration with *T*_R5–R7_. Oil and protein concentration data from rainfed regional studies in the US (Piper and Boote, 1999) and Argentina (Carrera et al., 2009) was obtained from equations reported in these studies. Oil and protein data from sunlit controlled environment chambers (Thomas et al., 2003; Alsajri et al., 2020) was extracted with PlotDigitizer v.3 (http://plotdigitizer.com, August 2, 2022). Meal protein concentration was estimated from oil and protein concentration based on equation 1 and expressed on a 12 mg g^–1^ moisture basis. Seed oil and protein concentration data is expressed on a 13 mg^–1^ moisture basis.

The variability in seed oil, protein, and estimated meal protein concentration explained by differences in T_*R*5–*R*7_ indicated that oil concentration increased with temperature following a quadratic response, with an optimum above the range of temperatures within our study (*p* < 0.001, partial *R*^2^ = 0.30, Figure 6A). Seed protein concentration also showed a quadratic response to T_*R*5–*R*7_, with an optimum temperature at 24°C, but with a low amount of the variability explained by temperature changes (*p* < 0.001, partial *R*^2^ < 0.01, Figure 6A). Meal protein concentration was better explained by changes in T_*R*5–*R*7_ (*p* < 0.001, partial *R*^2^ = 0.06) compared to seed protein concentration, and was highest at 27°C (Figure 6A). Overall, the cultivar random effect explained a similar amount of the variability in the analysis of seed oil and protein, and meal protein concentration in response to T_*R*5–*R*7_ (*p* < 0.001, partial *R*^2^ = 0.31–0.36; Figure 6A).

#### Cases with deficient meal protein concentration

Meal protein concentration is considered deficient when non-dehulled meal has a protein concentration below 440 mg g^–1^ (120 mg g^–1^ moisture basis). The estimated meal protein concentration fell below 440 mg g^–1^ in 105 cases or 7.1% of the treatments in our study (year, location, planting date, and cultivar combinations) and were the most frequent in College Station, TX, (16.3% of treatments at this location), followed by Columbia, MO, (13.3%), Rohwer, AR (7.8%), Milan, TN (7.0%), and Keiser, KY (5.7%). At other locations, meal protein concentration below 440 mg g^–1^ occurred in less than 5% of cases. In College Station, TX, most of the cases with low meal protein concentration occurred in 2013, and were found across different planting dates and MGs. In Columbia, MO, meal protein concentrations below the threshold were found most often with planting dates after May in 2014, and the frequency of low meal protein concentration increased with relatively longer MGs. Some of the low meal protein concentrations were associated with a cultivar effect. For instance, cultivars P5711RY and P6710RY grown during 2013 and 2014 had some of the highest frequencies of low meal protein concentration. Cultivar P39T67R, grown only in 2014, had the highest frequency of low meal protein concentration per year compared to other cultivars.

### Analysis of total oil and protein yields

**FIGURE 7:**
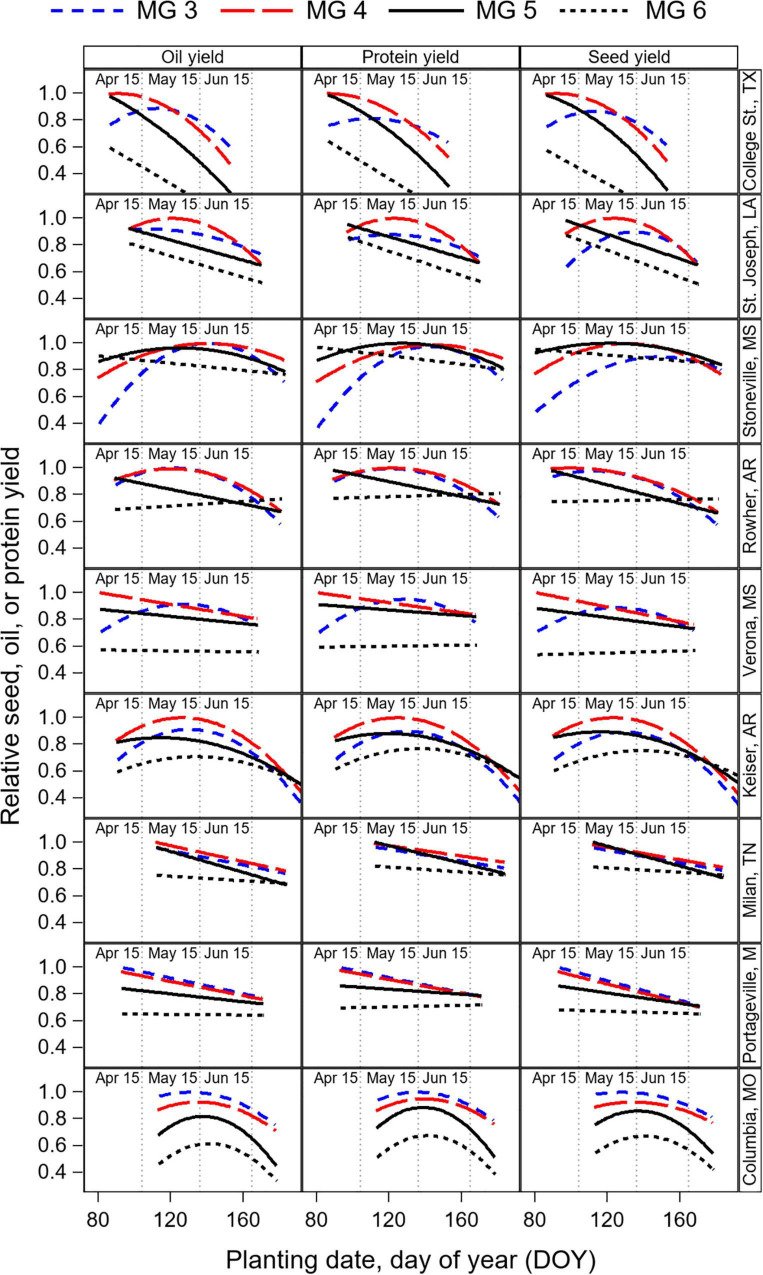
Relative seed, oil, and protein yield in response to PDOY by cultivar maturity group (MG) and by location, obtained from the quadratic model fit of the oil, protein, and seed yield response to planting date (Supplementary Tables 1–3). Data were averaged across years and cultivars within a MG before fitting the model, and relative yield normalized by the highest yielding cultivar MG within each location. Estimates of the parameters for the quadratic models, the root mean square error (RSME) from the residuals of the observed data with the model predictions, optimum PDOY to maximize yield, and estimated yield (in kg ha^–1^) at the optimum PDOY are provided in Supplementary Tables 1–3. The estimated relative seed yield, oil and protein yields by cultivar MG and location at planting dates on April 15, May 15, and Jun 15 are provided in Supplementary Table 4.

The response of total oil and protein yield to PDOY was analyzed with a quadratic or linear model depending on the MG and location that explained 65 and 64% of the total sum of squares in the model, respectively (Table 4). The shape of the response of seed yield, and total oil and protein yield to PDOY showed a quadratic relationship in 7, 6, 4 and 2 locations out of 9 for MG 3, 4, 5, and 6 cultivars, respectively (Figure 7 and Supplementary Tables 1–3). For the remaining location and MG combinations, seed yield, total oil and protein yield showed a linear response to PDOY, with the exception of MG 6 cultivars, for which the relationship was found not significant at *P* < 0.05 in three locations (Supplementary Tables 1–3). Based on the model fits obtained, the optimum planting date to maximize oil yield was on average sooner for MG 5 and 6 cultivars (April 11 and April 10, respectively) compared to MG 3 and 4 cultivars (April 27 and April 17) (Supplementary Tables 1–3). Similarly, the optimum planting dates to maximize protein yield occurred sooner for MG 5 and 6 cultivars on average across locations (April 13 and April 10), compared to MG 3 and 4 cultivars (April 29 and April 19, respectively) (Supplementary Tables 1–3). At locations and MG combinations where the seed yield, oil and protein yield showed a linear response to PDOY, the optimum planting date was the earliest within a location, and was equivalent to that for maximizing seed yield, oil, or protein yield (Supplementary Tables 1–3). However, when seed yield, oil and protein yield showed a quadratic response to PDOY, optimum planting dates to maximize oil and protein yield differed from those to maximize seed yield in some instances. For instance, the optimum planting date to maximize oil yield in MG 3 cultivars occurred 3, 1, 11, and 24 days earlier compared to optimum planting dates to maximize seed yield at Columbia, Verona, Stoneville, and St. Joseph, respectively (Supplementary Tables 1–3). Overall, optimum planting dates to maximize oil yield had a tendency to occur relatively earlier compared to those to maximize seed yield (4 days earlier on average across all locations and MG cultivars), and this was most evident for MG 3 cultivars (6 days earlier on average across all locations). Optimum planting dates to maximize protein yield occurred from 14 days earlier to 7 days later compared to those to maximize seed yield, but there was not a clear trend associated to cultivar MG (Supplementary Tables 1–3).

**TABLE 4:** Analysis of covariance for oil and protein total yields (kg ha^–1^), and seed yield (kg ha^–1^) with planting day of year (PDOY) as independent variable, and location (L), and maturity group (MG), allowed to affect the intercept, slope, and quadratic term of the regression.

		Oil yield (kg ha^–1^)			Protein yield (kg ha^–1^)			Yield (kg ha^–1^)		
Source	DF	*F*	*P*-value	% sum of squares	*F*-value	Pr > F	% sum of squares	*F*-value	Pr > F	% sum of squares
Location (L)	8	21.9	<0.001	19.2	21.88	<0.001	22.8	17.89	<0.001	22.7
MG	3	15.21	<0.001	13.0	11.2	<0.001	8.7	13.73	<0.001	9.3
L*MG	24	5.53	<0.001	3.9	5.7	<0.001	4.4	5.3	<0.001	5.4
PDOY	1	83.29	<0.001	6.8	112.73	<0.001	5.7	123.53	<0.001	4.1
PDOY*L	8	22.2	<0.001	3.7	22.56	<0.001	3.7	19.08	<0.001	3.1
PDOY*MG	3	22.77	<0.001	0.6	15.5	<0.001	0.5	18.29	<0.001	0.5
PDOY*L*MG	24	5.11	<0.001	2.1	5.21	<0.001	2.4	5.25	<0.001	2.6
PDOY*PDOY	1	141.25	<0.001	2.9	166.05	<0.001	3.1	174.81	<0.001	3.6
PDOY*PDOY*L	8	21.54	<0.001	1.2	21.39	<0.001	1.3	21.37	<0.001	1.5
PDOY*PDOY*MG	3	25.34	<0.001	1.4	16.61	<0.001	1.1	17.89	<0.001	1.2
PDOY*PDOY*L*MG	24	4.94	<0.001	0.8	5.04	<0.001	0.8	5.17	<0.001	1.0
Year (location)	14	49.63	<0.001	7.2	48.44	<0.001	6.4	38.93	<0.001	7.4
Cultivar(Year*MG)	42	10.14	<0.001	2.6	10.98	<0.001	2.9	7.17	<0.001	2.3
Residual	4554	–	–	34.6	–	–	36.2	–	–	35.3

Year nested within location was included as random factor in the model. Data were averaged across cultivars within a MG before analysis.

Overall, our results show that the response of protein yield to PDOY was similar to that of seed yield (Figure 7). Thus, cultivar MG recommendations to maximize protein yield were consistent with those to maximize seed yield based on the model estimates for planting dates on April 15, May 15, and June 15 (Supplementary Table 4). In contrast, the oil yield response to planting date revealed a relative advantage of short-season MG cultivars for oil yield production compared to full-season MG 5 and 6 cultivars. As a result, cultivar MG recommendations to maximize oil yield differed from those to maximize seed yield in some instances (Supplementary Table 4). For instance, for planting dates on May 15 and June 15 at Columbia and College St., yields were maximized with MG 3 and 4 cultivars, but recommendations to maximize oil yield would be MG 3 cultivars. At Stoneville, MG 4 and 5 cultivars maximized seed yield production for planting dates on May 15, and MG 3 to 4 cultivars maximized oil yield. When planting date was delayed to June 15, the advantage of using short-season MG cultivars for oil yield production was also evident. At Milan, Stoneville, and Keiser, MG 3 to 5 cultivars maximized seed yield for planting dates on June 15, but only MG 3 and 4 cultivars maximized oil yield. For early planting dates on April 15, the relative advantage of short-season MG 3 cultivars was less evident due to the pronounced quadratic yield response to planting date found at most locations for this cultivar maturity. For planting dates on April 15, only at St. Joseph, LA cultivar MG recommendations changed from MG 4 and 5 for seed yield, to MG 3–5 to maximize oil yield (Supplementary Table 4).

On average across all locations, cultivar MG 4 were the highest yielding for planting dates on April 15 (relative yield = 0.95), followed by MG 5 (0.90), and MG 3 (0.84). In contrast, to maximize oil yield for planting dates on April 15, cultivar MG 4 were the highest yielding (relative yield = 0.95), followed by MG 3 (0.89), and MG 5 (0.86). For planting dates on May 15, MG 4 cultivars maximize seed yield on average (relative yield = 0.90), followed by MG 3 (0.89), and MG 5 cultivars (0.82). To maximize oil yield production for planting dates on May 15, cultivar MG 3 were the best recommendation on average (relatively yield = 0.90), followed by MG 4 (0.89), and by MG 5 (0.78) (Supplementary Table S4).

## Discussion

The yield, oil, and seed protein concentration data from this study was previously analyzed as part of a larger national US dataset by Assefa et al. (2019), where environment (considered as the combination of location and planting date) explained as much as 74–85% of the variation in soybean seed oil and protein concentration, revealing the need to further understand how location and planting date interact to affect seed oil and protein concentration. In addition, protein concentration in soybean meal, rather than expressed on raw seed weight basis, is a trait of more interest for the industry that has received little attention. Our study is the first analysis of estimated meal protein concentration in non-dehulled soybean meal from a coordinated regional effort to understand the interactive effect of planting date and cultivar maturity across the US Midsouth. We found that the variation in seed oil concentration was largely explained by main effects of location and cultivar maturity (Table 2). In contrast, seed protein and meal protein concentration showed a large genotype by environment interaction, as well as variation due to year, and year by location (Figure 2). Thus, mean seed and meal protein concentration within a location were variable from year to year, as well as their response to planting date and cultivar maturity. Lastly, seed and meal protein concentration showed the largest percentage of variability associated to random factors and to unaccounted sources of variation in our analysis (Figure 2). Low repeatability in seed protein concentration is consistent with results by Dardanelli et al. (2006) and suggests that other site-specific factors may be driving variation in seed and meal protein concentrations, such as soil fertility and management practices that affect crop N availability (Bosaz et al., 2021; Chiluwal et al., 2021). However, we did find some significant effects and clear trends that provide new understanding on the interactive effect of planting date and cultivar maturity that can help increase oil yield and manage risk of deficient meal protein concentration.

### Planting date and cultivar maturity effect on seed oil concentration

We found that the variability in oil concentration across planting dates and cultivar MGs was partially associated with differences in *T*_*R*5–*R*7_ following a quadratic model (Partial *R*^2^ = 0.30, *p* = < 0.0001, Figure 6A). This quadratic response was consistent with results from other regional data analyses and studies under controlled conditions (Figure 6B), with some differences. In our study, oil concentration showed a less pronounced decline with decreasing *T*_*R*5–*R*7_, and a higher optimum T_*R*5–*R*7_ compared to previous regional studies under rainfed conditions (Piper and Boote, 1999; Carrera et al., 2009). These differences in model fit across regional studies in Figure 6B could be due to the warmer range of temperatures in our study compared to others. In addition, all sites in our study were irrigated to fulfill the crop evapotranspiration demand. It is possible that under irrigated conditions, the response of seed oil concentration to *T*_*R*5–*R*7_ is less pronounced and has a higher optimum *T*_*R*5–*R*7_ that maximizes oil concentration compared to previous rainfed studies. Relatively lower seed oil concentration in results from studies under controlled conditions in Figure 6B may be partially due to cultivar differences in seed oil concentration. In the study by Alsajri et al. (2020), the relatively low seed oil concentration may be caused by fertilizing plants with inorganic N fertilizer throughout the study, which likely increased seed protein concentrations (Figure 6B). Chiluwal et al. (2021) found that N fertilizer applications during seed growth increased seed protein and decreased seed oil concentration, compared to an unfertilized control.

Delays in planting date and cultivar maturity had a pronounced effect, reducing average *T*_*R*5–*R*7_, in particular at northernmost locations (Supplementary Figure 4). These results indicate that early planting dates and relatively short-season cultivar MGs are most critical at northernmost locations to avoid delays in the start of seed growth that cause reductions in *T*_*R*5–*R*7_ and seed oil concentration. The analysis of the combined planting date and cultivar maturity effect on the date of R5 (Figure 5) revealed that oil concentration decreased the most when date of R5 was delayed after July (by 0.39 mg g^–1^ day^–1^) in latitudes above 35°N (*p* < 0.001, *R*^2^ = 0.20). Overall, we found that the effect of delaying cultivar maturity on seed oil concentration (4.3 mg g^–1^ rMG^–1^, Figure 3) was greater on average compared to the effect of planting date (1.7 mg g^–1^ increase and 3.3 mg g^–1^ decrease in oil when advancing or delaying planting date of MG 4 cultivars, respectively, compared to planting dates in May; Figure 4). These results are in contrast with previous studies that found a minor effect of cultivar maturity on seed oil concentration relative to the effect of planting date (Mourtzinis et al., 2017; Assefa et al., 2019). This could be due to previous regional studies including cultivar maturities best adapted within a location and planting date, and less variation in cultivar maturities within the same environment.

### Planting date and cultivar maturity effect on seed and meal protein concentration

Previous studies found an inconsistent effect of planting date on seed protein concentration. Under irrigated conditions, delaying planting date had a positive or negative effect on seed protein concentration depending on the year in a study in Nebraska (Bastidas et al., 2008), no effect in Wisconsin (Pedersen and Lauer, 2003), an increase in seed protein concentration in Mississippi (Bellaloui et al., 2015), and no effect or a reduction in seed protein concentration in Arkansas (Bajaj et al., 2008; Jaureguy et al., 2013). Studies conducted under rainfed conditions at latitudes of 38.7°N and above found a positive or no effect of delay in planting date on seed protein concentration in most cases (Osler and Cartter, 1954; Helms et al., 1990; Kane et al., 1997; Pedersen and Lauer, 2003; Bajaj et al., 2008; Caviglia et al., 2011; Rowntree et al., 2013; Gaspar et al., 2017; Mourtzinis et al., 2017), with a negative effect on seed protein concentration found only by Robinson et al. (2009) in Indiana, and in the regional analysis by Assefa et al. (2019) at latitudes between 40 and 45°N. None of the abovementioned studies analyzed the effect of planting date on meal protein concentration.

The range of latitudes, planting dates, and cultivar maturities in our study were helpful to identify some patterns in the interactive effect of planting date and cultivar maturity on seed protein concentration (Figures 3–5). We found that planting dates before May were likely to decrease seed protein concentration (4.4 mg g^–1^ on average across locations and years for MG 4 cultivars; Figure 3), possibly due to a dilution effect of higher yields on average with planting dates before May, compared to planting dates in May. This hypothesis is supported by the optimum planting date of MG 4 cultivars found in our study (April 17 on average across locations; Supplementary Table 1), and by the well documented negative relationship between yield and seed protein concentration (Ortez et al., 2018; La Menza et al., 2019). When analyzing the effect of cultivar maturity, we found that switching to longer cultivar maturities increased seed protein concentration by 4.4 mg g^–1^ rMG^–1^ on average for planting dates before May, but to a lesser extent for planting dates in May (Figure 4).

For planting dates after May, the effect of planting date and cultivar maturity on seed protein concentration was variable depending on the location and year, with a trend that seemed associated to differences in latitude across locations (Figures 3, 4). We found that a greater part of the variation in seed protein concentration across planting dates and cultivar maturities was explained by day of year for start of R5 (*R*^2^ = 0.5–6%, Figure 5), compared to changes in *T*_*R*5–*R*7_ (partial *R*^2^ < 0.01, Figure 6A). In addition, meal protein concentration was better explained by changes in the start of R5 (*R*^2^ = 3–19%, Figure 5) and changes in *T*_*R*5–*R*7_ (partial *R*^2^ = 0.06) compared to variation in seed protein concentration. The relationship between seed protein concentration and *T*_*R*5–*R*7_ had been previously reported but often explains a low fraction of the variability in seed protein concentration, and can be inconsistent from one location to another, or from year to year (Figure 6B, Weiss et al., 1952; Howell and Cartter, 1958; Serretti, 1993; Kane et al., 1997; Naeve and Huerd, 2008). The negative quadratic model fit between seed protein concentration and *T*_*R*5–*R*7_ found in some studies (Figure 6B) may be an indirect result of the seed oil concentration response to *T*_*R*5–*R*7_ and the collinearity between seed protein and oil concentration. A regression analysis between meal protein and seed oil concentration concentration indicated low collinearity between these two variables in our data (*R*^2^ = 0.001, *p* = 0.006), compared to a more pronounced negative relationship found between seed protein and oil concentration (*R*^2^ = 0.17, *p* < 0.0001). We compared the shape of the relationship between meal protein concentration and *T*_*R*5–R7_ from a literature review of regional studies and studies under control conditions (Figure 6B), calculating meal protein concentration in other studies based on Equation 1. Overall, these results revealed a similar trend across most studies showing an increase in meal protein concentration with *T*_*R*5–*R*7_ (Figure 6B). It is possible that the relationships between seed and meal protein concentration in the study by Piper and Boote (1999) were partially affected by water stress, since it included data from southern (and northern) US variety test trials conducted during 1970 to 1990 without irrigation. The increasing trend in meal protein concentration with *T*_*R*5–*R*7_ may be explained by a negative effect of low temperatures on biological nitrogen fixation (Montañez et al., 1995), and by a reduction in the duration of seedfill and yield with increasing *T*_*R*5–*R*7_.

The estimated meal protein concentration decreased when the start of seedfill was delayed (Figure 5), and with decreasing *T*_*R*5–*R*7_ (Figure 6A), with relevant implications for northernmost locations in our study region. Previous studies reported a positive effect of delaying planting dates on seed protein concentration (Bellaloui et al., 2015; Mourtzinis et al., 2017); however, meal protein concentration was not reported in these studies. It is likely that late planting dates cause reductions in oil concentration due to lower *T*_*R*5–*R*7_ that would partially offset the benefit from high protein concentration in raw seed, and reduce meal protein concentration. Thus, our study suggests that quantifying the effect of planting date on seed protein concentration alone could be misleading when the goal is to increase meal protein concentration after oil extraction.

### Planting date by cultivar maturity strategies to increase meal protein concentration

The analysis of estimated meal protein concentration was helpful to identifying planting date and cultivar maturity choices that reduce risk of low meal protein concentration. The largest number of cases of deficient meal protein concentration in our study occurred with planting dates after May, and in particular for locations > 35°N. The number of cases with deficient meal protein concentration increased when the start of seed fill was delayed to mid-August with MG 5 and 6 cultivars (Figure 5). However, our results indicate it is possible to advance the start of the seed fill phase and reduce cases with insufficient meal protein concentration with MG 3 and 4 cultivars (Figure 5). Therefore, adapting cultivar MG choices to 3 and 4 when delaying planting date would decrease the risk of low meal protein concentration across the range of latitudes in our study, and is also consistent with optimum MG recommendations to maximize yield for late planting dates in the Midsouth based on Salmerón et al. (2016).

There is growing interest in early planting dates to increase soybean yields, but the effect of this management practice on seed and meal protein concentration has been less studied. We found that planting dates before May decreased the estimated meal protein concentration of MG 4 cultivars by 4.4 mg g^–1^ on average across site-years compared to planting dates in May (Figure 4). Cases of deficient meal protein concentration with early planting dates were most evident at latitudes < 35°N (Figure 5). Of interest, adapting to MG 5 cultivars would reduce the number of cases with deficient meal protein concentration with relatively early planting dates that cause the beginning of seed fill to occur before August 1, compared to using MG 3 and 4 cultivars (Figure 5). These results are consistent with the range of cultivar maturity recommendations that maximize yield with early planting dates in the same study (Salmerón et al., 2016).

### Cultivar maturity by planting date effect on total oil and protein yield

The total oil and protein yield, rather than the concentration, will be most important to consider an overall effect of the different management practices evaluated on the system’s productivity. Our goal was to quantify optimum planting date and MG recommendations that maximize oil and protein yield and compare if they differ from the management recommendations to maximize seed yield reported by Salmerón et al. (2016) based on the same dataset. We found that the response of total protein yield to planting date and cultivar maturity was similar to that of seed yield (Figure 7). In contrast, the oil yield response to planting date revealed an opportunity to increase oil yield with short-season MG cultivars. Thus, we found differences in the cultivar MG recommendations to maximize oil yield production compared to MG recommendations for seed yield at some locations for planting dates in May and after May (Supplementary Table 1). This was due to short-season cultivar maturities advancing the timing of reproductive stages, increasing *T*_*R*5–*R*7_ (Supplementary Figure 4), and increasing seed oil concentration by 4.3 mg g^–1^ rMG^–1^ on average in our study. For early planting dates on April 15, the relative advantage of short-season MG 3 cultivars was less evident due to the pronounced quadratic yield response to planting date found at most locations for this cultivar maturity. For planting dates on April 15, only at St. Joseph, LA cultivar MG recommendations changed from MG 4 and 5 for seed yield, to MG 3–5 to maximize oil yield.

## Conclusion

This study found that optimum planting date and cultivar maturity recommendations for the US Midsouth to maximize seed and protein yield are similar, but can differ from those to maximize total oil yield. Relatively short-season cultivars were beneficial to maximize oil yield for planting dates in May and June, but not for planting dates in April. Overall, there was large variability in seed protein concentration and in estimated meal protein concentration, unaccounted for by the genetics and management factors evaluated in this study, that may be associated to other site-specific factors such as management or soil characteristics. Early plantings before May had a tendency to decrease seed protein and estimated meal protein concentration, likely due to a dilution effect of high yields, but this effect was less evident in northernmost locations where low protein concentration is usually a limitation. We found that a combination of planting dates and cultivar maturity choices that targets the start of the seed fill phase before mid-August would lower the chances of deficient meal protein concentration for northernmost locations in our study. We found that conclusions based on the analysis of seed protein concentration were not always consistent with conclusions based on the analysis of estimated meal protein concentration. Estimated meal protein concentration showed reduced collinearity with oil concentration and is a trait of more interest for the food industry compared to seed protein concentration. Thus, the analysis of meal protein concentration may provide an advantage in future studies that analyze genetic, management, and environmental effects on seed protein concentration.

## Data availability statement

The original contributions presented in this study are included in the article/Supplementary material, further inquiries can be directed to the corresponding author.

## Author contributions

MS and LP contributed to conception and design of the study. FB, NB, LE, FF, BG, DH, JL, AM, TM, CN, GS, TU, DV, EV, and WW collected the data. MS organized the database, performed the statistical analysis, and wrote the first draft of the manuscript. EG contributed to the statistical analysis. LP, FF, and EV contributed to the critical manuscript revision. All authors contributed to the article and approved the submitted version.

## Abbreviations

*T*_*R*5–*R*7_: average daily air temperature from R5 to R7 soybean developmental stages.
