# Supplementary material for: Regional analysis of planting date and cultivar maturity recommendations that improve soybean oil yield and meal protein concentration
Source: Front Plant Sci. 2022 Oct 17;13:954111. doi: 10.3389/fpls.2022.954111 (PMC9618690; doi:10.3389/fpls.2022.954111)
Supplement: Supplementary file 1 [file Data_Sheet_1.docx]

**Supplementary Tables and Figures**

**Supplementary Table 1.** Estimates of the parameters for the quadratic (y=a +bx+cx^2^) models by location and cultivar maturity group (MG) that explain seed yield (y) as a function of planting day of year (PDOY, x), p-values for the quadratic and regression parameters being different than 0, root mean square error (RSME) from the residuals of the observed data with the model predictions, optimum PDOY to maximize yield, and estimated yield at the optimum PDOY. †Indicates when optimum planting date occurred at the earliest planting date within a location.

| **Location** | **MG** | **Parameter estimates** | | | | **P-value** | | **RMSE (kg ha^-1^)** | **Optimum PDOY** | **Max. yield**  **(kg ha^-1^)** |
| --- | --- | --- | --- | --- | --- | --- | --- | --- | --- | --- |
|  |  | **a** | **b** | **c** | **b** | | **c** |  |  |  |
| Columbia, MO | **3** | -1501 | 106.5 | -0.4135 | 0.027 | | 0.011 | 461 | 8-May | 5350 |
|  | **4** | -3032 | 118.7 | -0.4408 | 0.015 | | 0.007 | 506 | 14-May | 4953 |
|  | **5** | -14216 | 275.4 | -1.0079 | <0.001 | | <0.001 | 703 | 16-May | 4593 |
|  | **6** | -14769 | 263.1 | -0.9406 | <0.001 | | <0.001 | 521 | 19-May | 3635 |
| Portageville, MO | **3** | 6422.1 | -17.6 | - | <0.001 | | - | 661 | 2-Apr† | 4804 |
|  | **4** | 6141.7 | -16.3 | - | <0.001 | | - | 619 | 2-Apr† | 4641 |
|  | **5** | 4983.9 | -9.3 | - | <0.001 | | - | 462 | 2-Apr† | 4126 |
|  | **6** | 3439.1 | -1.9 | - | 0.334 | | - | 549 | 2-Apr† | 3266 |
| Milan, TN | **3** | 5308.7 | -10.2 | - | <0.001 | | - | 473 | 22-Apr† | 4167 |
|  | **4** | 5421 | -10.3 | - | <0.001 | | - | 579 | 22-Apr† | 4271 |
|  | **5** | 6135.7 | -16.1 | - | <0.001 | | - | 557 | 22-Apr† | 4335 |
|  | **6** | 4001 | -3.7 | - | 0.120 | | - | 575 | 22-Apr† | 3590 |
| Keiser, AR | **3** | -6787 | 169.6 | -0.6527 | <0.001 | | <0.001 | 721 | 9-May | 4236 |
|  | **4** | -4067 | 142.7 | -0.5774 | <0.001 | | <0.001 | 487 | 3-May | 4750 |
|  | **5** | 58.139 | 72.4 | -0.3129 | <0.001 | | <0.001 | 494 | 25-Apr | 4246 |
|  | **6** | -2391 | 86.4 | -0.3129 | <0.001 | | <0.001 | 674 | 18-May | 3576 |
| Verona, MS | **3** | -2910 | 114.2 | -0.4553 | <0.001 | | <0.001 | 542 | 5-May | 4254 |
|  | **4** | 5853.2 | -13.1 | - | <0.001 | | - | 527 | 21-Mar† | 4803 |
|  | **5** | 4899.9 | -8.3 | - | <0.001 | | - | 661 | 21-Mar† | 4233 |
|  | **6** | 2426.8 | 1.7 | - | 0.433 | | - | 850 | 21-Mar† | 2565 |
| Rohwer, AR | **3** | -579 | 97.2 | -0.4315 | <0.001 | | <0.001 | 632 | 22-Apr | 4891 |
|  | **4** | 2530 | 49.7 | -0.2495 | 0.012 | | <0.001 | 720 | 9-Apr | 5002 |
|  | **5** | 6445.3 | -17.4 | - | <0.001 | | - | 752 | 29-Mar† | 4917 |
|  | **6** | 3635.2 | 1.2 | - | 0.5217 | | - | 720 | 29-Mar† | 3739 |
| Stoneville, MS | **3** | -5142 | 126.2 | -0.4220 | <0.001 | | <0.001 | 972 | 29-May | 4298 |
|  | **4** | -2433 | 109.8 | -0.4184 | <0.001 | | <0.001 | 899 | 11-May | 4772 |
|  | **5** | 1766.6 | 49.7 | -0.2050 | 0.001 | | <0.001 | 1306 | 1-May | 4783 |
|  | **6** | 4983.8 | -5.2 | - | 0.004 | | - | 911 | 20-Mar† | 4571 |
| St. Josepth, LA | **3** | -13614 | 272.2 | -1.0063 | <0.001 | | <0.001 | 723 | 15-May | 4786 |
|  | **4** | -8059 | 216.6 | -0.8761 | <0.001 | | <0.001 | 608 | 3-May | 5334 |
|  | **5** | 7600.8 | -24.3 | - | <0.001 | | - | 671 | 6-Apr† | 5264 |
|  | **6** | 7255.8 | -26.8 | - | <0.001 | | - | 825 | 6-Apr† | 4680 |
| College St., TX | **3** | -4624 | 137.4 | -0.6042 | <0.001 | | <0.001 | 687 | 23-Apr | 3191 |
|  | **4** | -542.8 | 92.1 | -0.5010 | 0.004 | | <0.001 | 642 | 1-Apr | 3685 |
|  | **5** | 2407.9 | 44.3 | -0.3499 | 0.178 | | 0.009 | 761 | 26-Mar† | 3649 |
|  | **6** | 4484.8 | -27.7 | - | 0.001 | | 0.013 | 578 | 26-Mar† | 2134 |

**Supplementary Table 2.** Estimates of the parameters for the quadratic (y=a +bx+cx^2^) models by location and cultivar maturity group (MG) that explain oil yield (y) as a function of planting day of year (PDOY, x), p-values for the quadratic and regression parameters being different than 0, root mean square error (RSME) from the residuals of the observed data with the model predictions, optimum PDOY to maximize yield, and estimated yield at the optimum PDOY. †Indicates when optimum planting date occurred at the earliest planting date within a location.

| **Location** | **MG** | **Parameter estimates** | | | | **P-value** | | **RMSE (kg ha^-1^)** | **Optimum PDOY** | **Max. yield**  **(kg ha^-1^)** |
| --- | --- | --- | --- | --- | --- | --- | --- | --- | --- | --- |
|  |  | **a** | **b** | **c** | **b** | | **c** |  |  |  |
| Columbia, MO | **3** | -278.44 | 19.02 | -0.0758 | 0.021 | | 0.006 | 26.4 | 5-May | 915 |
|  | **4** | -415.04 | 19.20 | -0.0740 | 0.020 | | 0.008 | 26.4 | 9-May | 830 |
|  | **5** | -2103.26 | 42.18 | -0.1568 | <0.001 | | <0.001 | 40.7 | 14-May | 733 |
|  | **6** | -2116.21 | 38.73 | -0.1399 | <0.001 | | <0.001 | 28.8 | 18-May | 564 |
| Portageville, MO | **3** | 1113.12 | -3.10 | - | <0.001 | | - | 40.0 | 2-Apr† | 828 |
|  | **4** | 1058.25 | -2.87 | - | <0.001 | | - | 33.8 | 2-Apr† | 794 |
|  | **5** | 856.97 | -1.81 | - | <0.001 | | - | 27.2 | 2-Apr† | 690 |
|  | **6** | 574.82 | -0.50 | - | 0.158 | | - | 29.4 | 2-Apr† | 529 |
| Milan, TN | **3** | 1000.88 | -2.24 | - | <0.001 | | - | 27.8 | 22-Apr† | 750 |
|  | **4** | 1045.56 | -2.47 | - | <0.001 | | - | 30.5 | 22-Apr† | 769 |
|  | **5** | 1098.19 | -3.10 | - | <0.001 | | - | 30.5 | 22-Apr† | 751 |
|  | **6** | 692.38 | -0.77 | - | 0.058 | | - | 31.8 | 22-Apr† | 606 |
| Keiser, AR | **3** | -1146.7 | 29.13 | -0.1125 | <0.001 | | <0.001 | 37.9 | 9-May | 739 |
|  | **4** | -739.9 | 25.36 | -0.1027 | <0.001 | | <0.001 | 24.0 | 3-May | 825 |
|  | **5** | 26.3351 | 12.16 | -0.0539 | <0.001 | | <0.001 | 24.7 | 22-Apr | 712 |
|  | **6** | -376.97 | 14.23 | -0.0530 | <0.001 | | <0.001 | 30.7 | 14-May | 578 |
| Verona, MS | **3** | -372.62 | 17.55 | -0.0701 | <0.001 | | <0.001 | 32.6 | 5-May | 725 |
|  | **4** | 1016.98 | -2.34 | - | <0.001 | | - | 28.1 | 21-Mar† | 829 |
|  | **5** | 835.06 | -1.48 | - | <0.001 | | - | 36.1 | 21-Mar† | 716 |
|  | **6** | 447.31 | -0.02 | - | 0.964 | | - | 43.2 | 21-Mar | 446 |
| Rohwer, AR | **3** | -122.44 | 17.98 | -0.0804 | <0.001 | | <0.001 | 37.6 | 21-Apr | 883 |
|  | **4** | 510.16 | 8.20 | -0.0431 | 0.013 | | <0.001 | 39.8 | 5-Apr | 901 |
|  | **5** | 1129.41 | -3.16 | - | <0.001 | | - | 43.9 | 29-Mar† | 851 |
|  | **6** | 611.53 | 0.23 | - | 0.454 | | - | 38.5 | 29-Mar† | 632 |
| Stoneville, MS | **3** | -1939.12 | 40.75 | -0.1476 | <0.001 | | <0.001 | 31.4 | 18-May | 873 |
|  | **4** | -51.927 | 13.74 | -0.0527 | <0.001 | | <0.001 | 39.9 | 10-May | 844 |
|  | **5** | 251.57 | 10.00 | -0.0433 | 0.001 | | <0.001 | 63.7 | 25-Apr | 829 |
|  | **6** | 901.53 | -1.48 | - | <0.001 | | - | 37.2 | 20-Mar† | 785 |
| St. Josepth, LA | **3** | 136.6 | 13.20 | -0.0590 | 0.122 | | 0.052 | 39.4 | 21-Apr | 875 |
|  | **4** | -710.63 | 28.39 | -0.1212 | <0.001 | | <0.001 | 33.6 | 27-Apr | 952 |
|  | **5** | 1331.39 | -4.35 | - | <0.001 | | - | 38.4 | 6-Apr† | 914 |
|  | **6** | 1243.22 | -4.60 | - | <0.001 | | - | 46.2 | 6-Apr† | 801 |
| College St., TX | **3** | -1091.77 | 29.57 | -0.1296 | <0.001 | | <0.001 | 47.5 | 23-Apr | 595 |
|  | **4** | -79.0621 | 16.38 | -0.0903 | 0.003 | | <0.001 | 40.7 | 31-Mar | 664 |
|  | **5** | 616.88 | 4.37 | -0.0478 | 0.433 | | 0.036 | 49.9 | 26-Mar† | 643 |
|  | **6** | 827.58 | -5.23 | - | <0.001 | | - | 32.1 | 26-Mar† | 383 |

**Supplementary Table 3.** Estimates of the parameters for the quadratic (y=a +bx+cx^2^) models by location and cultivar maturity group (MG) that explain protein yield (y) as a function of planting day of year (PDOY, x), p-values for the quadratic and regression parameters being different than 0, root mean square error (RSME) from the residuals of the observed data with the model predictions, optimum PDOY to maximize yield, and estimated yield at the optimum PDOY. †Indicates when optimum planting date occurred at the earliest planting date within a location.

| **Location** | **MG** | **Parameter estimates** | | | | **P-value** | | **RMSE (kg ha^-1^)** | **Optimum PDOY** | **Max. yield**  **(kg ha^-1^)** |
| --- | --- | --- | --- | --- | --- | --- | --- | --- | --- | --- |
|  |  | **a** | **b** | **c** | **b** | | **c** |  |  |  |
| Columbia, MO | **3** | -945.8 | 39.03 | -0.1476 | 0.007 | | 0.002 | 52.7 | 12-May | 1634 |
|  | **4** | -1226.5 | 40.30 | -0.1478 | 0.005 | | 0.003 | 51.7 | 16-May | 1521 |
|  | **5** | -4277.3 | 83.85 | -0.3081 | <0.001 | | <0.001 | 72.5 | 16-May | 1427 |
|  | **6** | -4425.5 | 79.58 | -0.2855 | <0.001 | | <0.001 | 54.1 | 19-May | 1121 |
| Portageville, MO | **3** | 1979.8 | -5.49 | - | <0.001 | | - | 70.8 | 2-Apr† | 1475 |
|  | **4** | 1893.6 | -5.07 | - | <0.001 | | - | 61.6 | 2-Apr† | 1427 |
|  | **5** | 1501.8 | -2.64 | - | <0.001 | | - | 49.4 | 2-Apr† | 1258 |
|  | **6** | 1052.2 | -0.49 | - | 0.423 | | - | 54.3 | 2-Apr† | 1007 |
| Milan, TN | **3** | 1584.5 | -2.98 | - | <0.001 | | - | 48.1 | 22-Apr† | 1251 |
|  | **4** | 1562.8 | -2.68 | - | 0.000 | | - | 52.9 | 22-Apr† | 1263 |
|  | **5** | 1768.4 | -4.25 | - | <0.001 | | - | 54.4 | 22-Apr† | 1293 |
|  | **6** | 1247.5 | -1.33 | - | 0.061 | | - | 55.2 | 22-Apr† | 1098 |
| Keiser, AR | **3** | -1899.0 | 49.64 | -0.1922 | <0.001 | | <0.001 | 66.8 | 9-May | 1307 |
|  | **4** | -1068.4 | 41.79 | -0.1717 | <0.001 | | <0.001 | 42.5 | 1-May | 1474 |
|  | **5** | -88.9 | 23.78 | -0.1012 | <0.001 | | <0.001 | 46.9 | 27-Apr | 1308 |
|  | **6** | -892.4 | 29.00 | -0.1049 | <0.001 | | <0.001 | 58.1 | 18-May | 1112 |
| Verona, MS | **3** | -978.8 | 35.88 | -0.1412 | <0.001 | | <0.001 | 52.4 | 7-May | 1300 |
|  | **4** | 1708.0 | -3.53 | - | <0.001 | | - | 48.6 | 21-Mar† | 1425 |
|  | **5** | 1451.4 | -2.11 | - | 0.001 | | - | 64.1 | 21-Mar† | 1283 |
|  | **6** | 753.2 | 0.53 | - | 0.423 | | - | 75.9 | 21-Mar† | 795 |
| Rohwer, AR | **3** | 44.0 | 25.56 | -0.1150 | <0.001 | | <0.001 | 62.4 | 21-Apr | 1464 |
|  | **4** | 781.0 | 13.99 | -0.0692 | 0.015 | | 0.001 | 62.5 | 11-Apr | 1488 |
|  | **5** | 1930.9 | -5.02 | - | <0.001 | | - | 74.3 | 29-Mar† | 1489 |
|  | **6** | 1149.6 | 0.07 | - | 0.892 | | - | 64.9 | 29-Mar† | 1156 |
| Stoneville, MS | **3** | -3382.6 | 68.70 | -0.2428 | <0.001 | | <0.001 | 56.2 | 21-May | 1477 |
|  | **4** | -327.2 | 25.98 | -0.0941 | <0.001 | | <0.001 | 70.0 | 18-May | 1467 |
|  | **5** | 147.9 | 21.82 | -0.0881 | <0.001 | | <0.001 | 113.6 | 3-May | 1498 |
|  | **6** | 1612.2 | -2.24 | - | <0.001 | | - | 69.2 | 20-Mar† | 1435 |
| St. Josepth, LA | **3** | -405.3 | 30.67 | -0.1260 | 0.040 | | 0.018 | 66.2 | 1-May | 1461 |
|  | **4** | -1734.3 | 56.24 | -0.2331 | <0.001 | | <0.001 | 59.4 | 30-Apr | 1658 |
|  | **5** | 2339.4 | -7.42 | - | <0.001 | | - | 66.2 | 6-Apr† | 1627 |
|  | **6** | 2214.0 | -8.00 | - | <0.001 | | - | 78.6 | 6-Apr† | 1446 |
| College St., TX | **3** | -697.9 | 28.63 | -0.1274 | 0.003 | | 0.001 | 76.8 | 22-Apr | 910 |
|  | **4** | 249.6 | 20.14 | -0.1176 | 0.038 | | 0.003 | 66.0 | 26-Mar | 1112 |
|  | **5** | 742.8 | 12.44 | -0.0990 | 0.201 | | 0.013 | 86.6 | 26-Mar† | 1085 |
|  | **6** | 1465.3 | -9.09 | - | <0.001 | | - | 58.4 | 26-Mar† | 692 |

**Supplementary Table 4.** Relative seed, oil, and protein yield estimated by cultivar maturity group (MG), location, and planting date (April 15, May 15, or June 15). Values followed by different letters within a location, planting date, and variable (seed, oil, and protein yield) indicate different means at P<0.10.

|  |  | |  | |  | |  | |  |  | |  | |  | |  | |  |
| --- | --- | --- | --- | --- | --- | --- | --- | --- | --- | --- | --- | --- | --- | --- | --- | --- | --- | --- |
| **Cultivar MG** | **Relative Seed Yield** | | | | | **Relative Oil Yield** | | | | | **Relative Protein Yield** | | | | | |  |  |
|  | **Apr 15** | **May 15** | | **Jun 15** | | **Apr 15** | | **May 15** | | **Jun 15** | **Apr 15** | | **May 15** | | **Jun 15** | |  |  |
| Columbia, MO | | | | | | | | | | | | | | | | |  |  |
| **3** | - | 1.00 a | | 0.89 a | | - | | 0.99 a | | 0.86 a | - | | 1.00 a | | 0.90 a | |  |  |
| **4** | - | 0.93 a | | 0.84 a | | - | | 0.90 b | | 0.80 b | - | | 0.93 b | | 0.85 a | |  |  |
| **5** | - | 0.86 b | | 0.70 b | | - | | 0.80 c | | 0.63 c | - | | 0.87 b | | 0.70 b | |  |  |
| **6** | - | 0.68 c | | 0.56 c | | - | | 0.62 d | | 0.50 d | - | | 0.68 c | | 0.56 c | |  |  |
| Portageville, MO | | | | | | | | | | | | | | | | |  |  |
| **3** | 0.95 a | 0.84 a | | 0.73 a | | 0.95 a | | 0.84 a | | 0.72 a | 0.95 a | | 0.84 a | | 0.72 a | |  |  |
| **4** | 0.92 a | 0.82 ab | | 0.71 a | | 0.91 a | | 0.81 a | | 0.70 a | 0.92 a | | 0.82 ab | | 0.71 ab | |  |  |
| **5** | 0.83 b | 0.77 b | | 0.72 a | | 0.80 b | | 0.74 b | | 0.67 a | 0.83 b | | 0.77 b | | 0.72 a | |  |  |
| **6** | 0.67 c | 0.66 c | | 0.65 b | | 0.63 c | | 0.61 c | | 0.60 b | 0.68 c | | 0.67 c | | 0.66 b | |  |  |
| Milan, TN | | | | | | | | | | | | | | | | |  |  |
| **3** | - | 0.90 a | | 0.83 ab | | - | | 0.90 a | | 0.82 ab | - | | 0.91 a | | 0.84 ab | |  |  |
| **4** | - | 0.93 a | | 0.86 a | | - | | 0.92 a | | 0.83 a | - | | 0.93 a | | 0.87 a | |  |  |
| **5** | - | 0.91 a | | 0.80 ab | | - | | 0.88 a | | 0.76 b | - | | 0.92 a | | 0.82 ab | |  |  |
| **6** | - | 0.81 b | | 0.78 b | | - | | 0.76 b | | 0.73 b | - | | 0.82 b | | 0.79 b | |  |  |
| Keiser, AR | | | | | | | | | | | | | | | | |  |  |
| **3** | 0.81 c | 0.89 b | | 0.71 b | | 0.82 b | | 0.89 b | | 0.71 b | 0.82 c | | 0.88 b | | 0.7 b | |  |  |
| **4** | 0.96 a | 0.98 a | | 0.78 a | | 0.96 a | | 0.98 a | | 0.77 a | 0.97 a | | 0.98 a | | 0.77 a | |  |  |
| **5** | 0.89 b | 0.87 b | | 0.73 b | | 0.86 b | | 0.83 c | | 0.68 bc | 0.88 b | | 0.86 b | | 0.73 ab | |  |  |
| **6** | 0.69 d | 0.75 c | | 0.70 b | | 0.65 c | | 0.70 d | | 0.64 c | 0.68 d | | 0.75 c | | 0.70 b | |  |  |
| Verona, MS | | | | | | | | | | | | | | | | |  |  |
| **3** | 0.85 b | 0.88 a | | 0.73 a | | 0.84 b | | 0.86 a | | 0.73 a | 0.87 b | | 0.90 a | | 0.76 a | |  |  |
| **4** | 0.93 a | 0.85 a | | 0.76 a | | 0.93 a | | 0.84 a | | 0.76 a | 0.94 a | | 0.86 ab | | 0.79 a | |  |  |
| **5** | 0.84 b | 0.78 b | | 0.73 a | | 0.82 b | | 0.76 b | | 0.71 a | 0.86 b | | 0.82 b | | 0.77 a | |  |  |
| **6** | 0.54 c | 0.55 c | | 0.57 b | | 0.54 c | | 0.54 c | | 0.54 b | 0.57 c | | 0.58 c | | 0.59 b | |  |  |
| Rohwer, AR | | | | | | | | | | | | | | | | |  |  |
| **3** | 0.97 ab | 0.93 a | | 0.73 ab | | 0.98 a | | 0.93 a | | 0.72 a | 0.98 ab | | 0.94 a | | 0.75 b | |  |  |
| **4** | 1.00 a | 0.93 a | | 0.78 a | | 0.99 a | | 0.92 a | | 0.76 a | 1.00 a | | 0.94 a | | 0.80 a | |  |  |
| **5** | 0.92 b | 0.82 b | | 0.71 b | | 0.88 b | | 0.78 b | | 0.67 b | 0.94 b | | 0.84 b | | 0.74 b | |  |  |
| **6** | 0.75 c | 0.76 c | | 0.77 a | | 0.71 c | | 0.71 c | | 0.72 ab | 0.78 c | | 0.78 c | | 0.78 ab | |  |  |

**Supplementary Table 4 (cont.).** Relative seed, oil, and protein yield estimated by cultivar maturity group (MG), location, and planting date (April 15, May 15, or June 15). Values followed by different letters within a location, planting date, and variable (seed, oil, and protein yield) indicate different means at P<0.10.

| **Cultivar MG** | **Relative Seed Yield** | | | **Relative Oil Yield** | | | **Relative Protein Yield** | | |
| --- | --- | --- | --- | --- | --- | --- | --- | --- | --- |
|  | **Apr 15** | **May 15** | **Jun 15** | **Apr 15** | **May 15** | **Jun 15** | **Apr 15** | **May 15** | **Jun 15** |
| Stoneville, MS | | | | | | | | | |
| **3** | 0.73 c | 0.88 b | 0.87 ab | 0.83 b | 1.00 a | 0.87 ab | 0.78 c | 0.98 a | 0.89 a |
| **4** | 0.94 ab | 1.00 a | 0.89 ab | 0.93 a | 0.96 ab | 0.89 a | 0.91 b | 0.98 a | 0.93 a |
| **5** | 0.99 a | 0.99 a | 0.91 a | 0.94 a | 0.93 b | 0.82 b | 0.98 a | 0.99 a | 0.90 a |
| **6** | 0.93 b | 0.89 b | 0.86 b | 0.85 b | 0.80 c | 0.75 c | 0.92 b | 0.87 b | 0.83 b |
| St. Joseph, LA | | | | | | | | | |
| **3** | 0.74 c | 0.90 b | 0.72 a | 0.92 a | 0.88 b | 0.74 a | 0.86 bc | 0.87 b | 0.73 a |
| **4** | 0.95 a | 0.97 a | 0.71 a | 0.98 a | 0.95 a | 0.70 ab | 0.97 a | 0.97 a | 0.71 ab |
| **5** | 0.94 a | 0.80 c | 0.67 a | 0.91 a | 0.78 c | 0.64 b | 0.94 ab | 0.80 c | 0.67 b |
| **6** | 0.83 b | 0.68 d | 0.53 b | 0.79 b | 0.65 d | 0.50 c | 0.82 c | 0.68 d | 0.53 c |
| College Station, TX | | | | | | | | | |
| **3** | 0.86 b | 0.78 a | - | 0.88 b | 0.80 a | - | 0.81 b | 0.75 a | - |
| **4** | 0.97 a | 0.74 a | - | 0.97 a | 0.72 b | - | 0.96 a | 0.73 a | - |
| **5** | 0.86 b | 0.53 b | - | 0.82 c | 0.49 c | - | 0.85 b | 0.54 b | - |
| **6** | 0.42 c | 0.20 c | - | 0.41 d | 0.17 d | - | 0.45 c | 0.21 c | - |

**Supplementary Figure 1.** Mean effect of early (before May) and late (after May) planting dates by latitude and year on seed oil and protein concentration, and on meal protein concentration of MG 3 cultivars. Closed symbols indicate a significant planting date effect at P<0.05, obtained from the ANOVA analysis (Table 2).


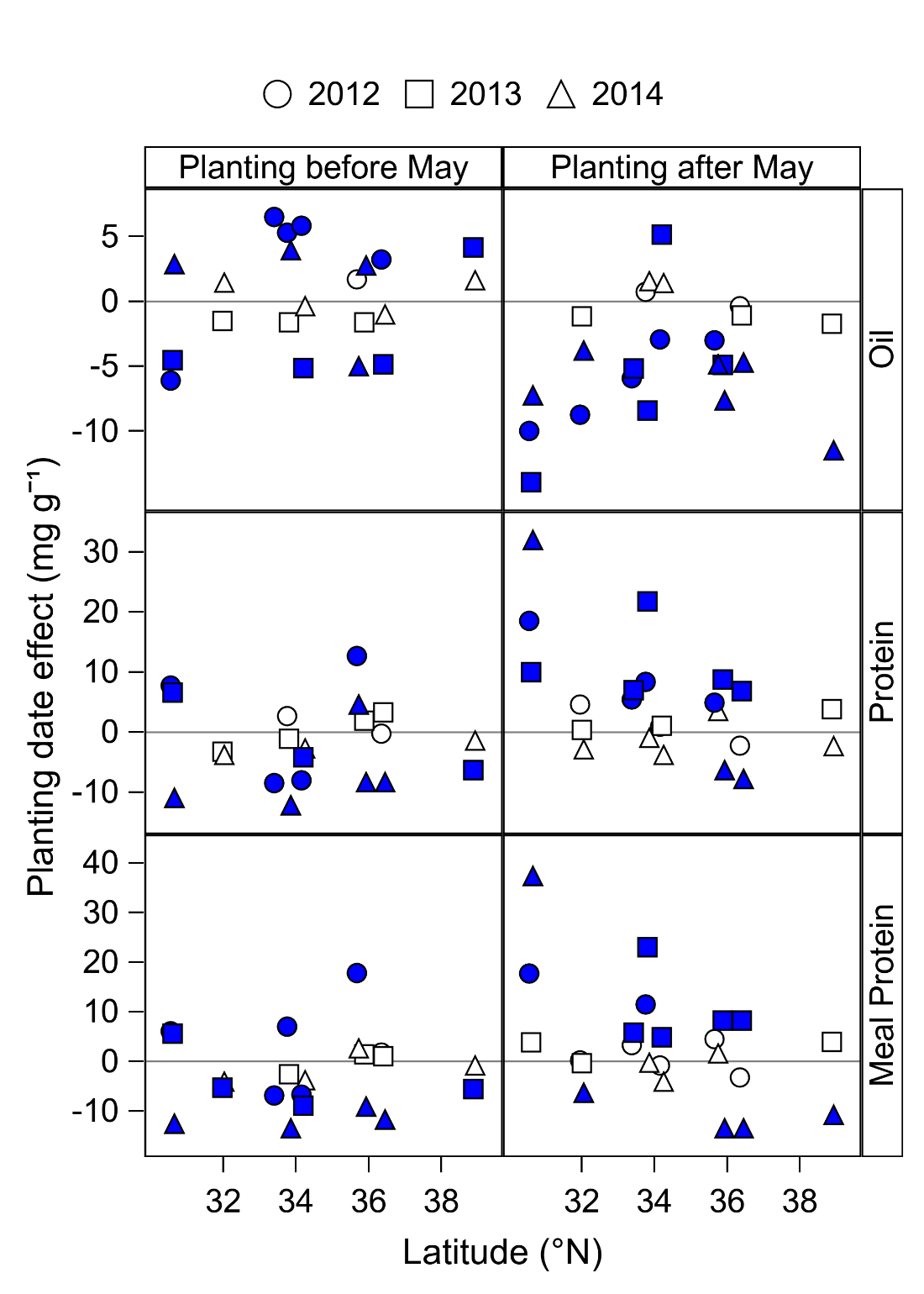


**Supplementary Figure 2.** Mean effect of early (before May) and late (after May) planting dates by latitude and year on seed oil and protein concentration, and on meal protein concentration of MG 5 cultivars. Closed symbols indicate a significant planting date effect at P<0.05, obtained from the ANOVA analysis (Table 2).


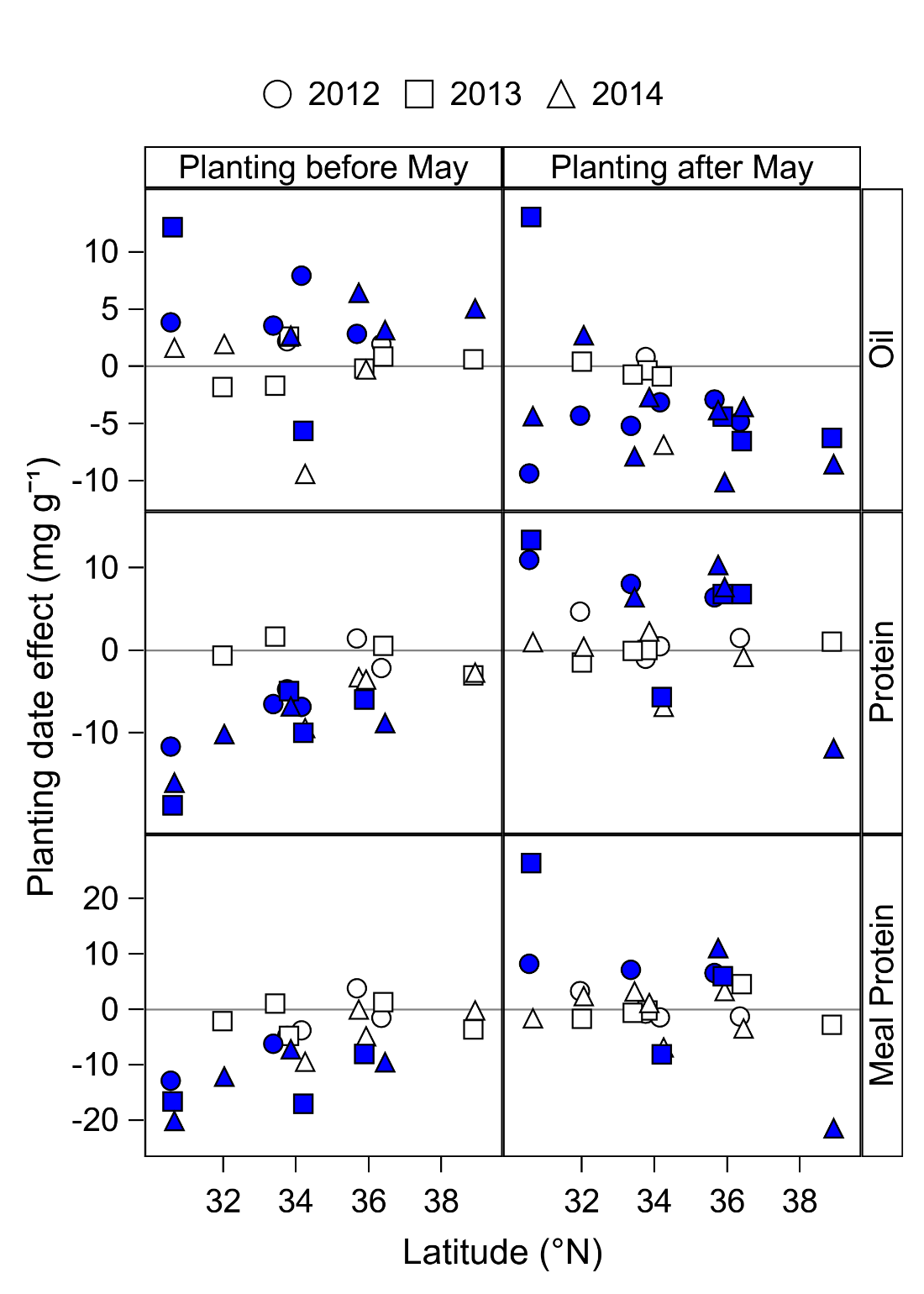


**Supplementary Figure 3.** Mean effect of early (before May) and late (after May) planting dates by latitude and year on seed oil and protein concentration, and on meal protein concentration of MG 6 cultivars. Closed symbols indicate a significant planting date effect at P<0.05, obtained from the ANOVA analysis (Table 2).


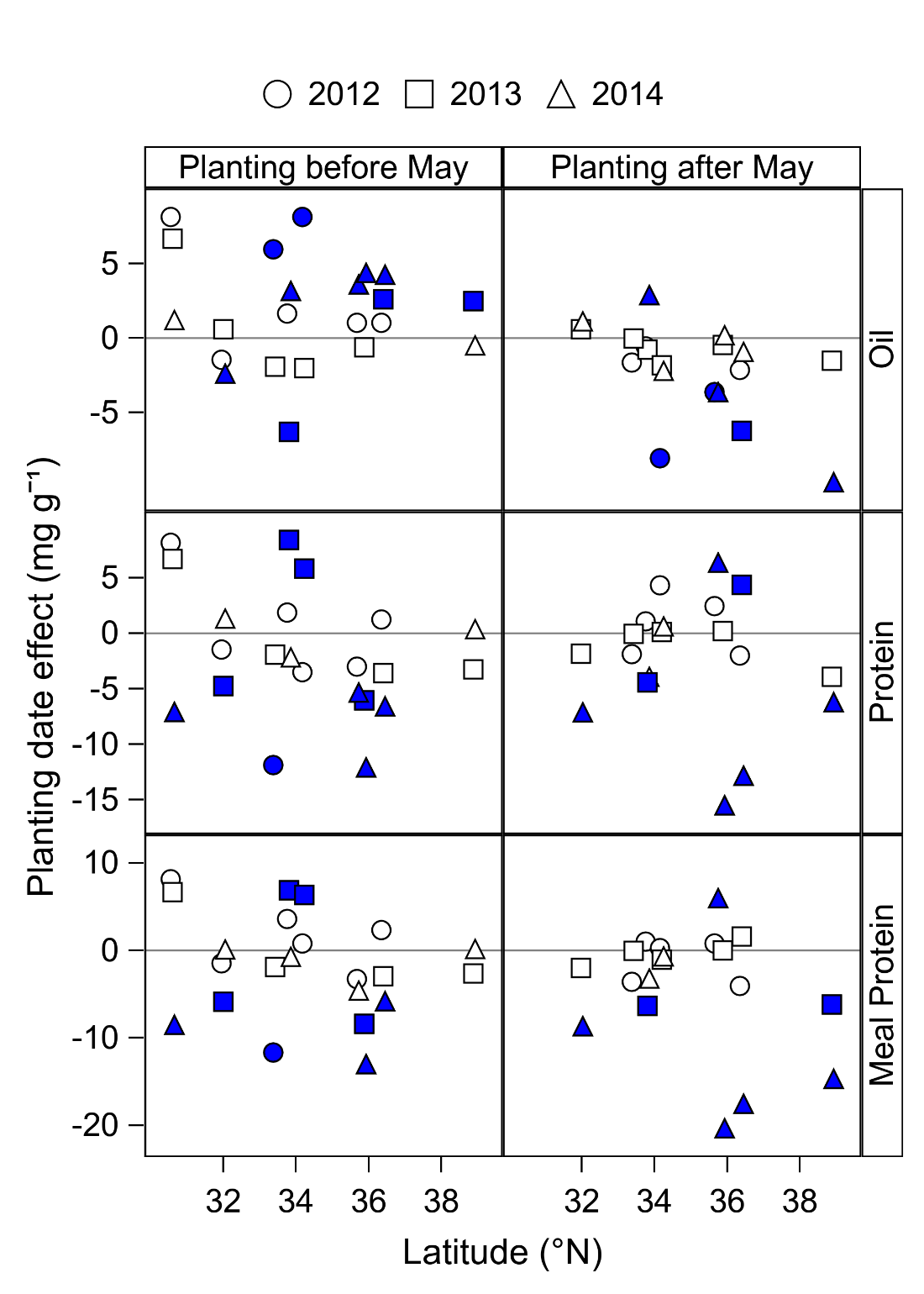


**Supplementary Figure 4.** Average temperature during seedfill (R3 to R5) by latitude and planting time (A) and by cultivar maturity group (B). Planting dates were grouped following Table 1 in planting dates before May (Early), planting dates in May, and planting dates after May (Late).

| 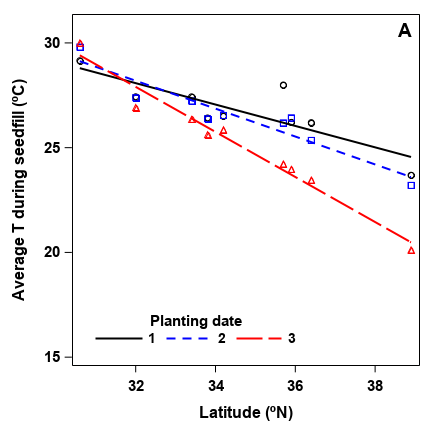 | 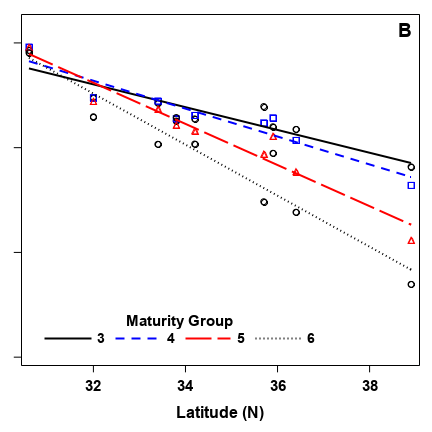 |
| --- | --- |
